# Study on effect of plant growth promoting rhizobacteria on sorghum (*Sorghum bicolor* L.) under gnotobiotic conditions

**DOI:** 10.3389/fmicb.2024.1374802

**Published:** 2024-10-04

**Authors:** M. Chiranjeevi, Geeta D. Goudar, Krishnaraj PU, Nagaraju Yalavarthi

**Affiliations:** ^1^Department of Agricultural Microbiology, College of Agriculture, Vijayapur, India; ^2^Department of Agricultural Microbiology, College of Agriculture, Dharwad, India; ^3^Central Silk Board, Central Sericultural Research and Training Institute, Berhampore, India

**Keywords:** antagonistic activity, IAA production, PGPR, siderophore production, sorghum, Bacillus subtilis, Open reading frame (ORF)

## Abstract

The rhizosphere is enriched with diverse microflora, allowing for delving prospective microorganisms to enhance crop growth and yield for varied soil conditions. Demand for millet growth-promoting microorganisms is a contemporary need for dryland agriculture. Therefore, a detailed survey was conducted in northern Karnataka, India, to identify the millet growing areas, particularly sorghum. The rhizobacteria from the sorghum (*Sorghum bicolor* L.) were assessed for promoting seed germination using the paper towel method and classified based on their efficiency. The elite isolates were positive for indole-3-acetic acid (IAA), gibberellic acid (GA), phosphate, zinc oxide solubilization, and hydrogen cyanide (HCN) production. The test isolates were antagonistic to *Macrophomina phaseolina* and *Fusarium* sp. and inhibited completely. Further evaluation of the cultures on sorghum growth-promoting attributes under pot culture conditions showed that the plants inoculated with PG-152 (*Bacillus subtilis*) recorded the highest plant height, chlorophyll content, root dry weight, shoot dry weight, and total dry weight under ideal conditions of fertilization. Two isolates, namely, PG-152 and PG-197, performing superior under pot culture conditions, were identified as *Bacillus subtilis* and PG-197 as *Enterobacter* sp., respectively, using 16S rDNA analysis. The sequences were allowed to screen open reading frames (ORF) and found several ORFs in *Enterobacter cloacae* and *Bacillus subtilis,* respectively. This study found that the rhizosphere is vital for identifying prospective isolates for biocontrol and plant growth-improving microorganisms.

## Introduction

Diversifying the cultivation of high-nutritive-value crops is in demand due to the global focus on nourishment and security (Carlson et al., 2020). Millets give product consumers various choices, including sustenance, food, and fodder. Of many millet crops, sorghum (*Sorghum bicolor* L. Moench), a resilient and climate-smart grain, is cultivated in arid and semi-arid areas of Africa and Asia and boosts agricultural productivity. It is a staple food that serves as a significant source of energy, protein, minerals, and vitamins for the world’s poorest people (FAO, 1995; Kumara et al., 2014) and serves as a feedstock for the manufacturing of ethanol (Wang et al., 2016). It was thus referred to as a “powerhouse of nutrition.” Millets are produced annually on 9.76 million hectares of land, with a total production of 13.21 million tons (USDA, 2019). India ranks third globally for sorghum production, however, experiencing a − 3.89% compound annual growth rate (CAGR) due to several abiotic and biotic factors (Chapke and Tonapi, 2019). Karnataka, Maharashtra, and Andhra Pradesh are the three states in India where sorghum is primarily grown; together, these three states produce 80% of the nation’s sorghum. Because of its wider adaptability to diverse habitats and its ability to grow in harsh conditions, sorghum is one of the essential commodities required for human existence (De Wet et al., 1976; Balakrishna et al., 2019). Sorghum is grown on 0.11 lakh hectares in Karnataka, where it produces 1.17 million tons and yields 3428 kg/hectare (Advanced estimates of food grains production 2021–2022, n.d.). Due to rainfed cultivation in India, the yield and productivity are comparatively poor to the rest of the globe (FAO, 2020). Food laws and subsidies impact India’s seed supply, output, and millet consumption, primarily encouraging rice and wheat consumption (Pandravada et al., 2013). However, recent initiatives from India, pushing the world for millet production and consumption, have tremendously impacted sorghum production and consumption.

Meanwhile, attention has been paid to the long-term viability of natural resources for future generations (Finkel et al., 2017). However, production constraints, such as abiotic and biotic stress, must be overcome to meet the current demand. The commercial practices of cultivation is intessive but exhaustive and environmentally harmful. Microorganisms naturally maintain the sustainability of resources, and the life that has historically existed on the earth either directly or tangentially nourishes them (Singh et al., 2017). Plant growth-promoting bacteria (PGPR), which impact the earth’s structural and functional health, is a broad name for the bio-stimulating organisms of soil. The community’s percentage of microbes that promote plant development ranges between 2 and 5% (Kloepper, 1978; Qessaoui et al., 2019). By supplying the plant with a bacterium-produced compound, such as plant growth regulators, facilitating the uptake of specific nutrients from the environment, or fixing the unavailable forms of nutrients, the PGPR microorganisms have a positive impact on seed germination, seed vigor, crop development, and improvement (Rahmoune et al., 2017). In addition to promoting rapid growth through the modulation of auxin phytohormones and reducing crop ethylene concentrations (Glick, 2012), or root-related nitrogen fixation (Döbereiner, 1992; Zarei et al., 2020), PGPR also promotes indirect plant growth when it acts on one or more phytopathogenic organisms and has detrimental effects on the pathogen through the production of antagonistic substances or the induction of pathogen resistance (Meena et al., 2020). To align the products with plant metabolism, they are triggered, controlled, and released at the expense of self-metabolism (Wortel et al., 2018). Commercial uses of the PGPR and how they work with plants offer tremendous hope for developing sustainable agriculture in the future.

Plant growth-promoting rhizobacteria (PGPR), such as *Bacillus* sp. and *Pseudomonas* sp., are helpful as agricultural biofertilizers because they can generate indole acetic acid (IAA) and gibberellins, which may improve plant development (Broughton et al., 2003). Veerubommu and Kanoujia (2011) claim that PGPR inoculations inhibit most pathogenic fungi-produced plant disease-causing organisms, protecting the plant from soil-borne illnesses (Lugtenberg and Kamilova, 2009). The increased use of biofertilizers, PGPR, and organic inputs reduces the dependence on synthetic inputs, such as insecticides and other additives. Overusing synthetic fertilizers has raised the cost of farming inputs, especially for small-scale farmers, and has had a detrimental effect on soil health and other critical environmental issues such as water and soil pollution and other health-related issues (Gupta et al., 2015). This situation highlighted the need for developing alternative production techniques that are less harmful to the ecosystem and more accountable for maintaining soil health. There is a knowledge deficit regarding the PGPR for sorghum products, focusing on promoting development and yield under organic cultivation. Therefore, this research aims to identify and extract helpful microorganisms for the growth of sorghum crops.

## Materials and methods

### Soil sampling and isolation of strains

In three northern Karnataka districts, namely, Bagalkote, Kalaburagi, and Vijayapura, during rabi in 2017–18, rhizosphere soil samples (*n* = 50) were obtained from crops including chickpea, pigeon pea, and maize. A total of 10 g of rhizosphere soil was transferred to a 250 mL conical flask containing 90 mL of sterile distilled water and vortexed for approximately 5 min. After that, 0.1 mL of the 10^−5^ and 10^−6^ dilutions were added to the respective media plate (Nutrient agar). The plates were placed in a BOD incubator and kept at 28°C for 48 h (Nagaraju et al., 2021), and the typical bacterial colonies were selected and purified by four-way streaking.

### Rapid screening of the native isolates by the seed germination test (the paper towel method)

The sorghum seeds (M-35-1, 70% germination percentage) were tested for germination using the paper towel technique indicated by ISTA guidelines (Anonymous, 1999). The test was performed using 100 seeds per blotting paper with three replications. In a nutshell, 100 sorghum seeds weighing on average 3.9 g were surface-sterilized with 0.1% HgCl_2_ (mercuric chloride, w/v) for 2 min, followed by three washes with sterile distilled water. The seeds were treated with test cultures of 24 h old with less than 10^9^ cfu/mL for 10 min. A reference strain (*Pseudomonas fluorescens*) was employed as a control. In the cabinet seed germinator, germination paper with treated seeds was incubated in the slanting position. The germination test was conducted at a constant temperature of 25 ± 2°C and a relative humidity of 95 ± 1% (%). The percentage (%) was calculated based on the number of germinated seeds. As a control, seeds were treated with sterile distilled water. After 7 days of seeding, the root length, shoot length, and germination percentage were measured (Dasgupta et al., 2015). Refer to Supplementary Table 1 for further information.


$$\mathrm{Germination}\left(\%\right)=\frac{\mathrm{Number}\;\mathrm{of}\;\mathrm{seeds}\;\mathrm{germinated}}{\mathrm{Number}\;\mathrm{of}\;\mathrm{seeds}\;\mathrm{put}\;for\;\mathrm{germination}}\times 100$$

### Root, shoot lengths (cm), and vigor index (VI)

After the seventh day of incubation, five seedlings were randomly chosen from each treatment. Root and shoot lengths were measured using a gradient scale from the tip of the primary root to the base of the hypocotyl, and the shoot length was measured from the tip of the primary leaf to the base of the hypocotyl (cm). For measures of root and shoot length, five healthy seedlings were chosen at random. The vigor index was determined by multiplying the germination percentage by the average root and shoot lengths (Abdul and Anderson, 1973).


$$\begin{array}{l} \mathrm{Vigor}\;\mathrm{Index}\;\left(VI\right)=\left(\mathrm{Mean}\;\mathrm{root}\;\mathrm{length}+\mathrm{Mean}\;\mathrm{shoot}\;\mathrm{length}\right)\\ \times \mathrm{percentageof}\;\mathrm{germination}\text{.} \end{array}$$

### Functional characterization

Based on the profile of the potential to enhance seed germination, the five best test isolates were chosen for further research. The colonies were moved to nutrient agar slants and were morphologically and biochemically characterized (Cappuccino and Sherman, 1992); the details may be found in Supplementary Tables 2, 3. Under *in vitro* circumstances, the properties of the five rhizosphere microorganisms, namely, PG-145, PG-148, PG-152, PG-178, and PG-197 that promote plant development were assessed. The production of indole acetic acid (Gordon, 1951), gibberellic acid (Paleg, 1965), P-solubilization, potassium release, Zn solubilization (Nagaraju et al., 2017, 2021), and HCN production (Castric and Castric, 1983) are all included in the functional characterization.

### The indole-3-acetic acid production test

The IAA generation of test isolates was assessed using active cultures that had existed for 24 h. The test isolates were grown separately in tubes filled with 5 mL of nutrient broth and incubated for the specified time and temperature. These cultures underwent a 10-time, 10,000 rpm centrifugation procedure after incubation. A 2 mL of Salkowski reagent was added to the supernatant, and the development of color was regarded as positive for IAA generation after 30 min of incubation.

### Estimation of gibberellic acid

A test tube containing 2 mL of zinc acetate and 25 mL of the fresh culture supernatant was used. After 2 min, 2 mL of potassium ferrocyanide was added, followed by a 15-min centrifugation at 10,000 rpm. Then, 5 mL of this supernatant was mixed with 5 mL of 30% hydrochloric acid, and the mixture was then incubated at 20°C for 75 min. Next, 5% HCl was used to treat the control sample. In a UV–Vis spectrophotometer, the absorbance of the samples and the control was measured at 254 nm (Paleg, 1965). The gibberellic acid concentration was determined using a standard curve and was expressed in g/ml of medium.

### The phosphate solubilization test

The broth culture was centrifuged in a centrifuge tube at 10,000 rpm for 10 min to separate the supernatant from the cell mass and insoluble phosphate (tricalcium phosphate) (Pikovskaya, 1948). The phosphomolybdic blue color technique (Jackson, 1973) assessed the amount of soluble P in the supernatant by measuring the blue color using a spectrophotometer at 610 nm after 15 min. After 5 days of inoculation, the quantity of Pi released in the broth was calculated compared to a control group that had not been inoculated.

### The potassium releasing test

The isolates were tested for their capacity to release potassium from insoluble potassium sources, such as mica (alumino silicate), added to Aleksandrov’s medium. A 10 μL spot of overnight-grown culture was added to each plate, and it was then incubated at 28 ± 2°C for 2 to 3 days (Nagaraju et al., 2017). The isolates with a distinct solubilization zone surrounding the colony were identified as potassium solubilizers. The observations were made up to 7 days after inoculation.

### The zinc solubilization test

The test isolates were examined using the Tris minimum medium supplemented with 0.1% zinc oxide to determine their capacity to solubilize the inorganic zinc from the zinc oxide. (ZnO, calamine, or zinc white). Then, 10 μL of the test isolates’ overnight-grown culture were spotted onto the solid medium. Zinc was considered soluble in the isolates, with a clear zone surrounding the colony on the medium (Nagaraju et al., 2017). We measured and indicated the diameter of the solubilization zone in mm.

### Nitrogen estimation from a bacteria

The N_2_-free malate broth was used to test the strains’ ability to fix nitrogen. A total of 1 mL of culture that had been grown for 24 h was added to the broth, and it was then incubated at 37°C for 7 days. The culture was homogenized, and 10 mL of it was digested using 5 mL of concentrated H_2_SO_4_ and 0.2 g of a K_2_SO_4_: CuSO_4_: selenium (100, 10:1) digestion catalyst combination. After cooling, distilled water was added to bring the level to 10 mL. Later, 20 mL of 40% NaOH was added to an aliquot of 10 mL, then transferred to a micro-Kjeldhal distillation unit, and distilled. Then, 0.066 g of bromocresol green and 0.033 g of methyl red were added to 100 mL of methanol, and a 4% boric acid mixed indicator was used to trap ammonia until the solution changed pink to green. To assess the total nitrogen content of the culture, it was titrated against 0.01 N H_2_SO_4_; the findings were represented as mg N_2_ fixed per g of malate (Ashraf et al., 2011).


$$\mathrm{Percent}\;N=\frac{\mathrm{Titer}\;\mathrm{value}\times 0.014\times N\;\mathrm{of}\;\mathrm{sulfuric}\;\mathrm{acid}\times \mathrm{vol}.\mathrm{made}\;}{\mathrm{The}\;\mathrm{volume}\;\mathrm{of}\;\mathrm{sample}\;\mathrm{used}}\times 100$$

### The HCN production test

The hydrogen cyanide (HCN) test was carried out using nutritional agar plates with 3% NaCl. Each test isolate’s pure culture was injected in 1 mL portions on individual media plates. A disk of Whatman filter paper No. 1 impregnated with an alkaline picric acid solution (0.5% picric acid (w/v) in 1% sodium carbonate) and sized to fit a Petri plate was placed in the upper lid of the inoculated while maintaining aseptic conditions. The plates were wrapped with parafilm to avoid the losses of HCN, and the control plate did not receive the inoculum. The plates were incubated at 28 ± 2°C for 48–72 h in a BOD incubator. HCN production was assumed to be indicated by a change in color from yellow to light brown, moderate, or reddish solid brown (Castric and Castric, 1983). Each experiment was repeated three times, and controls were kept.

### The siderophore test

Following the instructions provided by Schwyn and Neilands (1987) and Wei et al. (1991), respectively, siderophore production was determined. The overnight-grown cultures were inoculated into the chrome azurol S (CAS) agar media, and 10 μL of each culture was spotted on the media plates. The corresponding plates were incubated at 28 ± 2°C for 48 h. The formation of an orange-colored zone around the colony was considered positive for siderophore production. The orange zone’s diameter was measured for the qualitative evaluation.

### Antagonistic activity of the isolates

The PGPR strains were evaluated *in vitro* using a dual culture approach described by Sakthivel and Gnanamanickam (1987) against two specific sorghum crop diseases, namely, *Macrophomina phaseolina* and *Fusarium* sp. The fungi were first cultivated on potato dextrose agar (PDA) plates until they completely covered the medium surface. Using a sterile cork borer, the fungal cultures (10 mm diameter) were transferred on fresh PDA plates and developed for up to 48 h. The test isolate was then parallel streaked over both sides of the fungal disk, leaving a 1.5 cm space from the plate’s border. The PDA dishes that had only been inoculated with pathogens served as the appropriate controls. The dishes underwent an additional 96-h incubation period at 30°C. The fungal growth diameter was measured on the control and test culture inoculated plates. The zone of inhibition (ZOI) of each fungal pathogen by different isolates was calculated using the following formula:


$$\begin{array}{l} ZOI=\mathrm{Colony}\;\mathrm{diameter}\;\left(\mathrm{control}\;\mathrm{plate}\right)\\ -\mathrm{Colony}\;\mathrm{diameter}\;\left(\mathrm{in}\;\mathrm{dual}\;\mathrm{inoculated}\;\mathrm{plates}\right) \end{array}$$

$$I=\frac{C-T\;}{C}\times 100$$

where,

I is the percent inhibition, C is the radial growth of the fungal pathogen in control, and T is the radial growth of the pathogen in treatment.

### Pot culture evaluation of elite rhizobacterial isolates on Sorghum

The efficacy of five effective plant growth-promoting bacterial isolates, namely, PG-145, PG-148, PG-152, PG-178, and PG-197, on the growth and yield of sorghum was examined in a pot culture experiment carried out during rabi in 2019–20. For the pot experiment, the University of Agricultural Sciences, Dharwad’s College of Agriculture, provided the black cotton soil (regur), which was then taken from there. The soil was placed in plastic containers and autoclaved at 121°C for 1 h at a weight of 15 lbs. (9 kg) (30 cm top diameter). The sorghum seedlings (var. M-35-1, duration: 125–135 days) were treated with the appropriate strains; more information on the treatment is provided in Supplementary Table 4. The inoculum production for seed bacterization was performed in a nutrient broth medium, and the broth was inoculated with the appropriate strains and incubated for 3 days at 28 ± 2°C. After being properly combined with talc powder in a 1:3 ratio, the cultures were air-dried for an entire night in a laminar airflow chamber before being utilized to treat the seeds (Weller and Cook, 1983).

Five treated seeds were planted in each pot when the treated seeds were planted. Following germination, trimming was done to keep just one plant in each pot, and constant watering was done throughout the experiment to keep the soil at the ideal moisture level. After 30, 60, and 90 days of planting, measurements, such as plant height, root and shoot dry weights, chlorophyll index (using a SPAD meter), and total dry weight, were recorded. Following the crop’s harvest, the grain yield was measured. The recommended dose of fertilizers for sorghum is 50:25:00 kg NPK per hectare, which was applied as urea and single superphosphate (SSP) to each treatment. The required quantity of fertilizer per pot was calculated, and 0.33 g of urea and 0.12 g of SSP were added to each pot to supply 50:25 Kg of N:P_2_O_5_ (per ha) on a soil weight basis as per the package of practices.

### Molecular characterization of the isolate

The isolates with growth-promoting attributes under pot culture conditions were further characterized using the 16S rDNA analysis. For this, the strains were grown 24 h in nutrient broth at 30°C in a BOD incubator. The genomic DNA of the rhizobacterial isolates and reference strains was isolated by following Sambrook and Russell (2001), and the 16S rRNA gene was amplified with a set of universal primers 27F (5’AGAGTTTGATCCTGGCTCAG3`) and 1492R (5’TACGGYTACCTTGTTACGACTT3’). The reaction mixture details are given in Supplementary Table 6. The quality of the DNA was evaluated using agarose gel electrophoresis. Later, polymerase chain reaction (PCR) was performed with 100 ng template DNA, 5 U Taq DNA polymerase, 1 mM dNTP, 10 pM primer each, and 25 mM MgCl_2_ to make up a final volume of 20 μL (The reaction mixture details are given in Supplementary Table 5). Amplification was done in an Eppendorf Thermal Cycler (Supplementary Table 6). PCR-amplified DNA fragments of 1.3 kb products were analyzed for 16S rDNA sequencing using the Sanger sequencing method. The resulting contiguous DNA sequences were compared with the other sequences in the NCBI database using the Basic Local Alignment Search Tool (BLAST). The strains’ closest 16S rDNA gene sequences were retrieved from the www.ncbi.nlm.nih.gov database and aligned using Clustal_W, and the phylogenetic tree was constructed using the UPGMA method with MEGA X. The nucleotide sequences were deposited in the GenBank, and the accession numbers were obtained.

### Statistical analysis

The data were analyzed in Minitab v.19.2, and the results were interpreted by following the guidelines of Pansey and Sukhatme (1985). The level of significance used in the F- and *t*-tests was *p* = 0.01. Critical difference values were calculated wherever the F-test values were significant. The molecular data were analyzed using MEGA-XI and Split Plot software.

### Accession numbers of the isolates

Based on the BLAST similarity percentage, the isolates were identified as *Bacillus subtilis* (PG-152) and *Enterobacter* sp. (PG-197). The sequences were submitted to the NCBI GenBank with accession numbers CP0211231.1 and KY930712.1.

## Results

### Isolation of rhizosphere microorganisms

**Figure 1:**
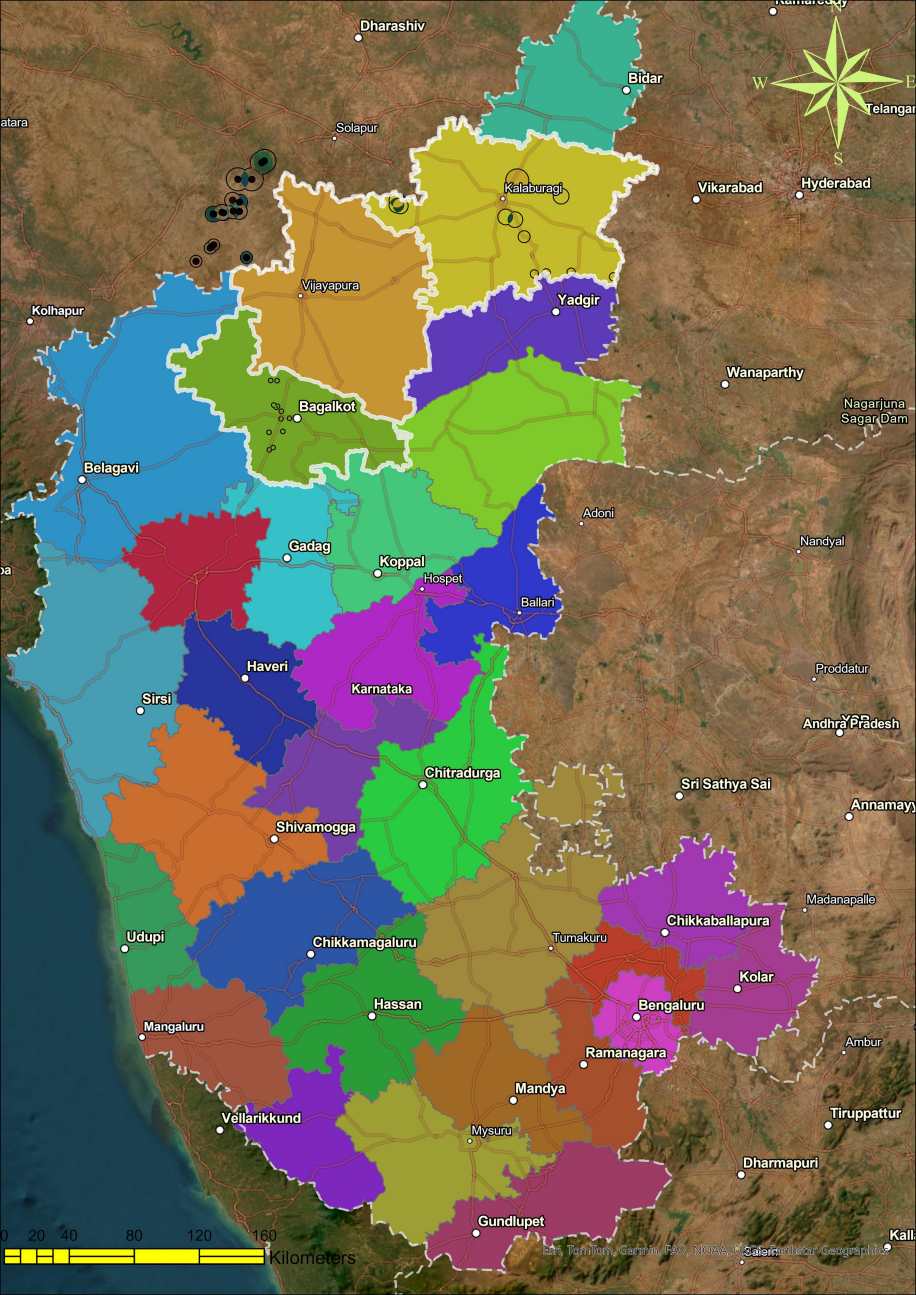
Soil sampling site: map depicting the soil sampling sites in Northern Karnataka, a total of three districts were covered during the survey and sampling (borders of Maharashtra and Karnataka, India, were also included for sampling).

In northern Karnataka, specifically Bagalkote, Kalaburagi, and Vijayapura, a comprehensive and detailed study was carried out during rabi in 2017–18, and 50 samples of chickpea, pigeon pea, and maize rhizospheres were gathered (Figure 1). In total, 209 strains were isolated based on the variations in colony morphology. Following three additional purifications to validate strains, careful choices of various colonies were made based on the colony’s color, structure, and surface. According to purity, the strains were further identified into 197 isolates, each tested *in vitro* for its ability to improve sorghum seed germination under controlled conditions.

### Seed bio-priming and germination tests

**Figure 2:**
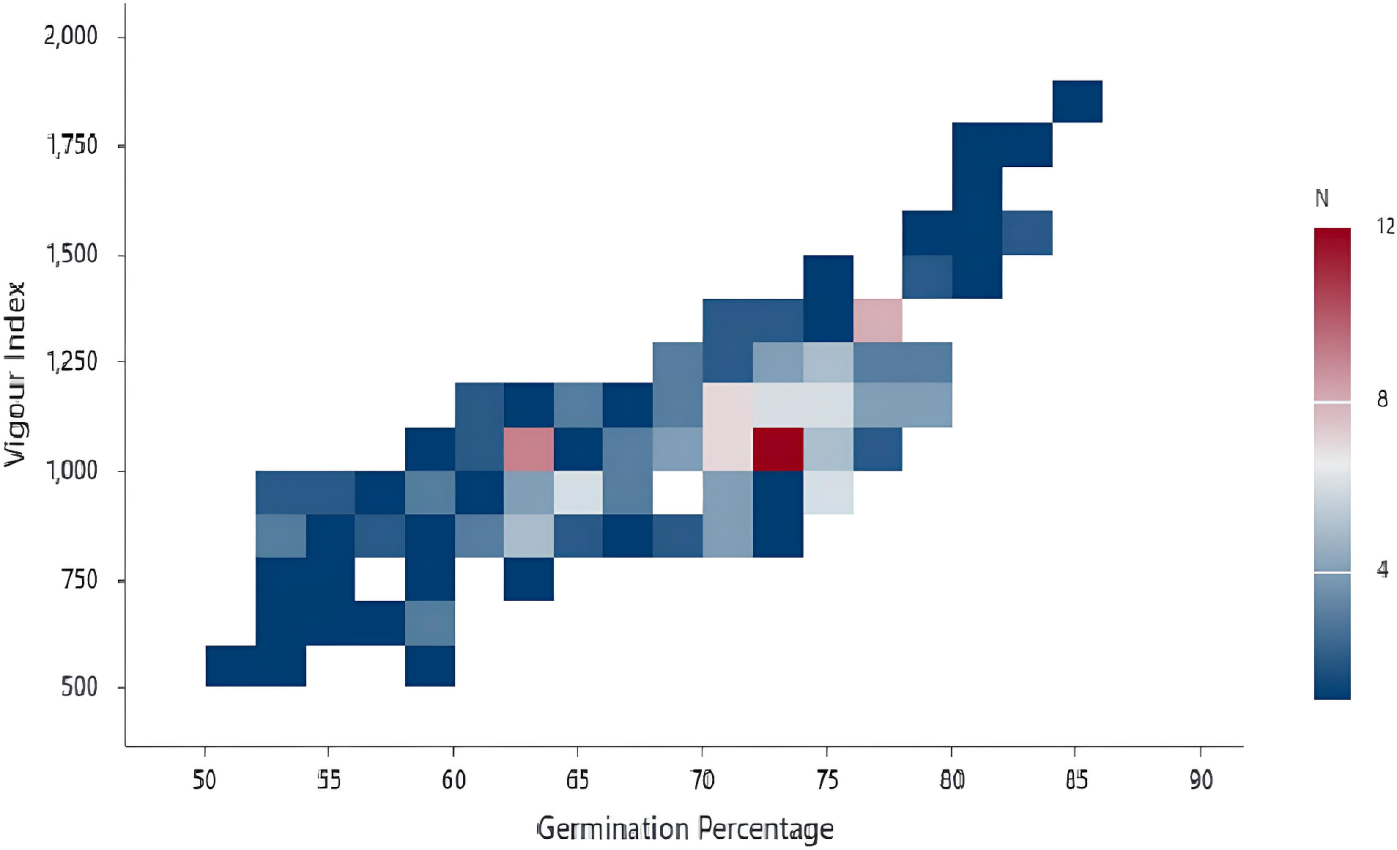
Binned plot depicting sorghum seed germination and vigor index: the sorghum seed was treated with the inoculum of bacterial isolates and evaluated for the seed germination and vigor index (*N* = number of treatments).

**Figure 3:**
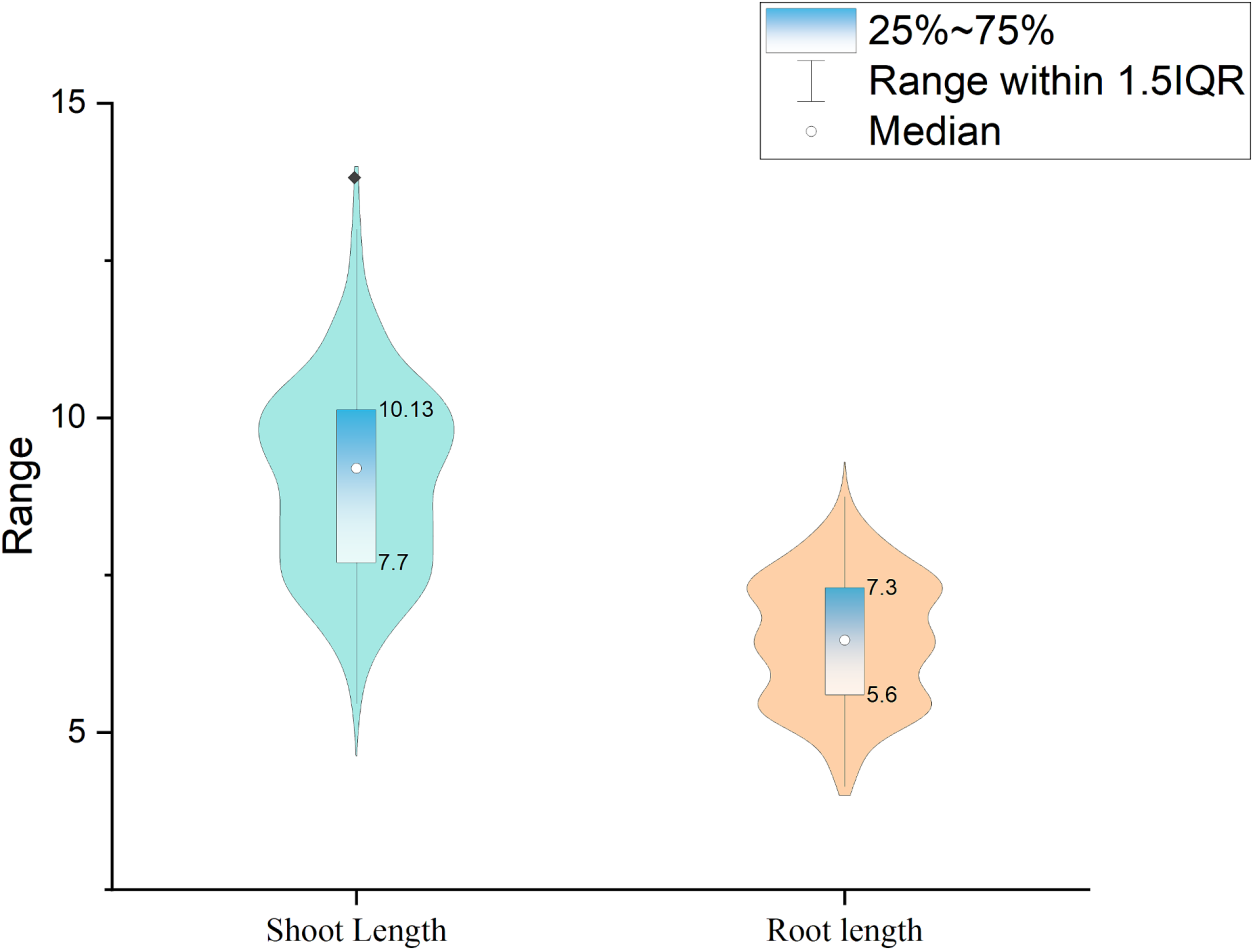
Violin plot representing the shoot length and root lengths: the germinated seeds were measured for their shoot and root lengths. The violin plot represents the number of samples, and the box and whisker represent the maximum and minimum values.

One of the main criteria for classifying the isolates as plant growth-promoting isolates is improving seed germination. Therefore, screening for isolates with improved seed germination abilities gained significance among the screening techniques. The current study employed 197 isolates, one reference, and one control for seed bio-priming (water treatment). *Pseudomonas fluorescens* treatment improved the germination percentage, which was 3.5% higher than the seeds treated with the PG-152 and PG-145 test isolates, which had encouraging germination rates of 82.5%. For the remaining isolates, the seeds germinated at a rate ranging from 85 to 50%, with a median of 70.50%. The examination of root length revealed that the longest root measured 8.32 cm, 5% shorter than the plants immunized with the reference strain (8.75 cm). The average shoot length in plants inoculated with PG-152 was 6% (or 13 cm) shorter than in plants inoculated with the reference strain (13.82 cm). Later, the seed vigor analysis was 5.6% lower than the plants vaccinated with the reference strain (1873.31). The control treatment results showed that, in comparison with effective culture treatments, the water treatment had little impact on improving seed germination. Based on the seed germination percentage and seed vigor index, five strains were selected for further studies, such as PG-145, PG-148, PG-152, PG-178, and PG-197. The results are presented in Figures 2, 3 and Supplementary Table 1.

### Morphological and biochemical characteristics

The colony morphology of three isolates—PG-145, PG-148, and PG-152—and the reference strain (*Pseudomonas fluorescens*) was found to be round, whereas other strains produced colonies with irregular shapes. All colony features were examined and documented. In instances PG-145 and PG-152, the colony was milky white, whereas, in cases PG-178 and PG-197, it was yellowish. Both the *Pseudomonas fluorescens* reference strain and PG-148’s colonies were white. PG-145 had elevated colonies, PG-148, PG-152, and the reference strain had flat colonies, and PG-178 and PG-197 had convex colonies, according to reports on colony elevation. For PG-148, PG-178, and PG-197, the colony consistency was smooth; however, for PG-145 and PG-152, it was dry. Undulated colony margins were seen in the strains PG-145 and PG-152 but not in the reference strains PG-148, PG-178, or PG-197 (*Pseudomonas fluorescens*). Except for PG-152, all of the cells showed Gram negative responses and were rod-shaped, according to the measurements of cell morphology at the microscopic level. Endospore production was observed only in PG-152 (Supplementary Table 2).

The outcomes of the biochemical tests revealed heterogeneity. All isolates and reference strains passed the ammonia synthesis, citrate utilization, and oxidase tests but failed the indole production and lactose fermentation tests. Other isolates, including the reference strain (*Pseudomonas fluorescens*) other than PG-145, demonstrated positive starch hydrolysis. Except for PG-178, other isolates and reference strains (*Pseudomonas fluorescens*) were positive for casein hydrolysis and nitrate reduction. Except for two isolates (PG-178 and PG-197), other isolates and reference strains showed positive for gelatin hydrolysis, catalase, and urease tests. The information is given in Supplementary Table 3.

### Plant invigorating attributes

Most rhizosphere microbes create phyto-regulatory substances, which aid plants in coping with challenging situations and sustaining a healthy life cycle. These include screening for the elite isolates with indole acetic acid (IAA), gibberellic acid (GA), hydrogen cyanide (HCN), siderophores, phosphorous, zinc, and potassium solubilization. Tryptophan-fortified broth to estimate indole-3-acetic acid is produced by the test isolates, ranging from 12.00 to 20.17 g/mL. IAA production is present in all isolates, with *Pseudomonas fluorescens* (the reference strain) and PG-152 reporting the highest levels of IAA production (*Bacillus subtilis*).

All the strains, including the reference strain, produced gibberellic acid under *in vitro* conditions ranging from 0.42 to 2.69 μg/mL of broth. The phytohormone production was reportedly high in the isolate PG-152 (*B. subtilis*) strain with 2.14 μg/mL of broth under optimal growth conditions. In the phosphate solubilization test, all the isolates showed solubilization zones of phosphate on Pikovskaya’s agar medium. The zone of solubilization ranged from 3 to 7.5 mm, which falls under the FCO 1985 commercial biofertilizers requirements. The isolate PG-197 (*Enterobacter* sp.) showed the maximum zone of P solubilization (7.5 mm) and released the highest amount of P_i_ (4.45%) from TCP broth. The amount of inorganic phosphate (P_i_) released from tricalcium phosphate in Pikovskaya’s broth after 5 days of inoculation (DAI) was quantified (Table 1). The percent P_i_ released by test isolates ranged from 0.91 to 4.45%. Among the isolates, PG-197 (4.45%) released the highest amount of inorganic phosphate (*p* < 0.01), followed by isolate PG-178 (4.3%). The amount of P_i_ released by the reference strain (*Pseudomonas fluorescens*) was 4.05%, and the lowest amount of P_i_ was released by the PG-145 (0.91).

**Table 1:** Functional characterization of native rhizobacterial isolates.

Sl. No.	Isolates	IAA (μg ml^−1^ of broth)	GA (μg ml^−1^ of broth)	Zone of P solubilization (Dia in mm)	Percent Pi released in broth	Zone of Zn solubilization (Dia in mm)	HCN production	Siderophore
1	PG-145	12.00	0.42	3.00	0.91	5.0	−	−
2	PG-148	13.81	1.07	6.00	3.40	3.0	+	+
3	PG-152	18.49	2.14	6.2	3.84	6.0	++	+
4	PG-178	17.26	1.28	7.0	4.30	3.0	+	+
5	PG-197	16.35	2.04	7.5	4.45	4.0	−	+
6	Reference strain (*P. fluorescens*)	20.17	2.69	6.5	4.05	3.0	+++	+
S.Em ± CD @ 1%		0.24	0.08					
		0.77	0.24					

*percentage of Pi released from Pikovskaya’s broth. −, indicates no zone of solubilization. +, positive for the test with minimal production. ++, moderate production. +++, high production.

In the zinc oxide solubilization test, strains showed Zn solubilization zones ranging from 2 to 6 mm, wherein strain PG-152 exhibited a maximum zone of solubilization (6 mm). The strains were also positive for HCN production but were limited to three. Among them, reference strain produced intense (+++) HCN, PG-152 was scaled as moderate (++), and PG-148 and PG-178 were scored as weak HCN producers based on the brown color production (Table 1). All the rhizobacterial isolates were tested for the production of siderophores, which was identified by the production of brown to orange color on chrome azurol S (CAS) agar media. All the isolates showed positive test results, including the reference strain (*P. fluorescens*). In the potassium-releasing test, none of the isolates tested positive.

### Antagonistic activity of rhizobacteria against phyto fungal pathogens

The dual culture method was used to test the strains’ hostile behavior. The five top rhizobacterial strains were evaluated against the *Macrophomina phaseolina* and *Fusarium* sp. fungi. The screening findings revealed that the isolates tested positively for the test with varying levels of pathogen percent inhibition above control. From 58.56 to 85.13%, less *M. phaseolina* mycelial growth was inhibited compared to the control. The Bacillus subtilis strains PG-148 and PG-152 showed the inhibition of Fusarium Sp. culture under invitro conditions, the inhibition percentages were 85.56 and 93.33% respectively. One native isolate, PG-152 (*B. subtilis*), showed competitive inhibition of 85.56, 8.7% less than the reference strain (93.33%).

### Sorghum pot experiment

**Figure 4:**
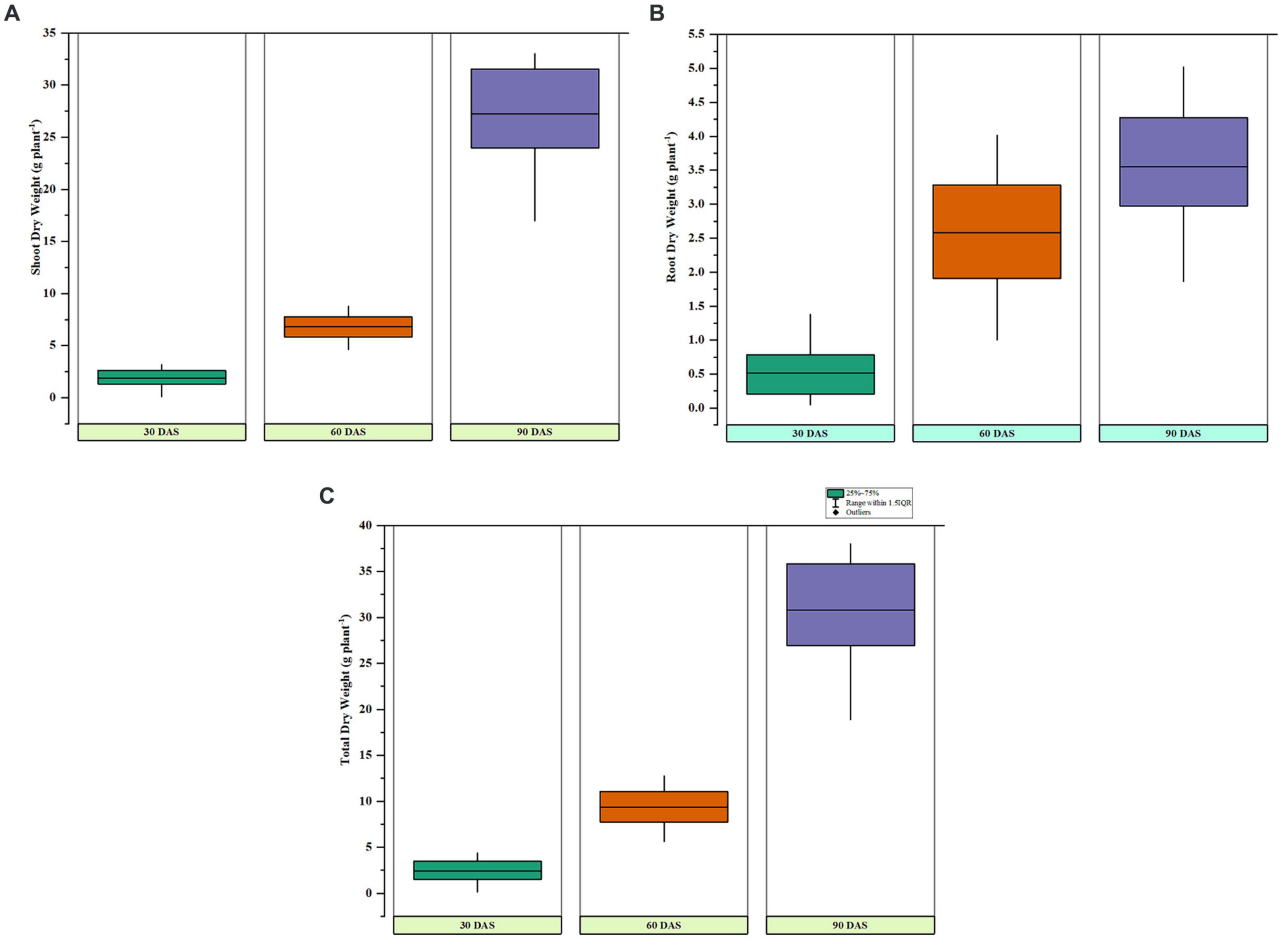
Box and whisker plots representing **(A)** dry shoot weights, **(B)** root dry weights, and **(C)** total dry weights of sorghum at 30, 60, and 90 days after sowing under pot culture conditions.

Under pot culture, the five top isolates and one reference strain were examined for the traits that encourage plant development. During the growth of the crop, several points were used to record features such as shoot and root length (Plant height). With the reference strain (*P. fluorescens*) treatment, the shoot length was at its maximum after 30, 60, and 90 days of seeding. The longest shoot measured in plants treated with the reference strain after 90 days of germination was 161.20 cm. The strain PG-152 (*B. subtilis*) treatment showed a commensurate shoot length during 30, 60, and 90 DAS, and the shoot lengths of 32.90, 83.00, and 143.20 cm were reported, respectively. The control has the most diminutive plant height throughout the experiment (*p* > 0.01). Correspondingly, the dry shoot weights were maximum with *P. fluorescens* treatment at 60 and 90 DAS; meanwhile, *B. subtilis* treatment showed an effect during initial (30 DAS) dry weights. The maximum dry weights were reported as 3.19, 8.76, and 33.3 g/plant at 30, 60, and 90 days after sowing. The root dry weights were significantly (*p* < 0.01) higher in reference-treated plants than in the other treatments. The highest root dry weight of 1.38 g/plant was recorded in *P. fluorescens* treatment at 30, 60, and 90 days after sowing, followed by PG-152-treated plants (1.02 g/plant) at 30 DAS. The dry root weights reported at 60 and 90 days after sowing were 4.02 and 5.02 g/plant with the reference strain. The strain PG-152 also showed statistically significant (*p* < 0.01) root dry weights after 60 and 90 days, which were recorded as 3.54 and 4.7 g/plant, respectively (Figure 4). The total dry weight of 38.03 g/plant (90 DAS) was recorded as the highest with the treatment of *P. fluorescens* (the results are presented in Table 2).

**Table 2:** Biometric and yield observations of sorghum pot experiment.

Treatments	Plant height (cm)			Chlorophyll content (SPAD value)			Shoot dry weight			Root dry weight			Total dry weight			Grain yield (g/plant)	N and P uptake (g/plant)	
	30 DAS	60 DAS	90 DAS	30 DAS	45 DAS	60 DAS	30 DAS	60 DAS	90 DAS	30 DAS	60 DAS	90 DAS	30 DAS	60 DAS	90 DAS		N	P
T_1_: PG-145	27.46	72	131	22.5	32	42.2	1.44	6.16	25.52	0.24	1.96	3.02	1.68	8.12	28.54	34.58	0.45	0.33
T_2_: PG-148	28.18	76.6	132.8	26.14	32.92	43.98	2	6.7	27.33	0.26	2.31	3.3	2.26	9.01	30.63	35.16	0.53	0.34
T_3_: PG-152	32.9	81.4	152.4	25.52	36.58	47.56	3.19	8.2	32	1.02	3.54	4.7	4.21	11.74	36.7	39.72	0.64	0.41
T_4_: PG-178	31.88	77.2	134.6	23.5	33.76	43.7	2.04	7.28	29.76	0.46	2.93	3.7	2.52	10.21	33.46	35.4	0.54	0.36
T_5_: PG-197	32.68	80.64	143.2	24.86	34.56	44.52	2.18	7.31	31.06	0.54	3.02	3.85	2.73	10.33	34.91	36.12	0.63	0.43
T_6_: Reference strain (*P. fluorescens*)	34.46	83	161.2	26.42	35.72	45.52	3	8.76	33	1.38	4.02	5.02	4.38	12.78	38.02	41.62	0.57	0.4
T_7_: RDF	26.46	69.2	121	20.16	29	40.07	1.17	5.5	22.4	0.17	1.86	2.93	1.34	7.36	25.33	33.36	0.37	0.32
T_8_: Control	20.66	47	103.8	18	20.8	30.08	0.08	4.6	17	0.05	1	1.86	0.13	5.6	18.86	29.32	0.21	0.15
S.Em ±	0.69	0.75	0.96	0.5	0.64	0.67	0.03	0.07	0.43	0.007	0.04	0.04	0.07	0.07	0.21	0.13	0.01	0.01
CD @ 1%	1.79	2.19	2.79	1.46	1.89	1.97	0.07	0.22	1.24	0.02	0.13	0.12	0.02	0.23	0.61	0.39	0.03	0.03

The rhizobacterial isolate-infused treatments considerably increased the chlorophyll content compared to the absolute control. The plants’ chlorophyll concentration was noted at 30, 45, and 60 DAS. Interestingly, even with 60 DAS after bacterial inoculation, the chlorophyll content in the plants was still at its highest, reaching a maximum of 47.56 SPAD in the PG-152-treated plants. The treatment of *P. fluorescens* at 30 DAS revealed a chlorophyll concentration of 26.42 SPAD value, indicating that the original chlorophyll content was constant. The exemplary control had the lowest level of chlorophyll (18.00 SPAD value). At 45 DAS, the highest chlorophyll content of 36.58 SPAD value was recorded with PG-152, and on-par results were observed in the *P. fluorescens*-treated plants with 35.72 SPAD value. The highest grain yield of sorghum observed was 41.62 g plant^−1^ in *P. fluorescens-*treated plants, which were significantly superior over all other treatments. The subsequent highest grain yield of 39.72 g plant^−1^ was recorded with the PG-152-treated plants. Plant phosphorus (P_i_) and nitrogen (N_2_) absorption were measured at harvest time. The plants treated with PG-152 absorbed most nitrogen (0.64 g plant^−1^), followed by those treated with PG-197 (0.63 g plant^−1^). The reference strain (*P. fluorescens*)-treated plants showed 0.57 g plant^−1^ N uptake, and the control plants showed negligible N uptake (0.21 g plant^−1^). The P absorption peaked in the PG-147-treated plants and was measured at 0.43 g plant^−1^. The PG-152-treated plants and 0.41 g plant^−1^ and 0.40 g plant^−1^ were reported as having on-par P uptake. Comparing treatments, absolute control had the lowest P uptake (0.15 g plant^−1^).

### Sequence alignment, identification, and ORF finding

**Figure 5:**
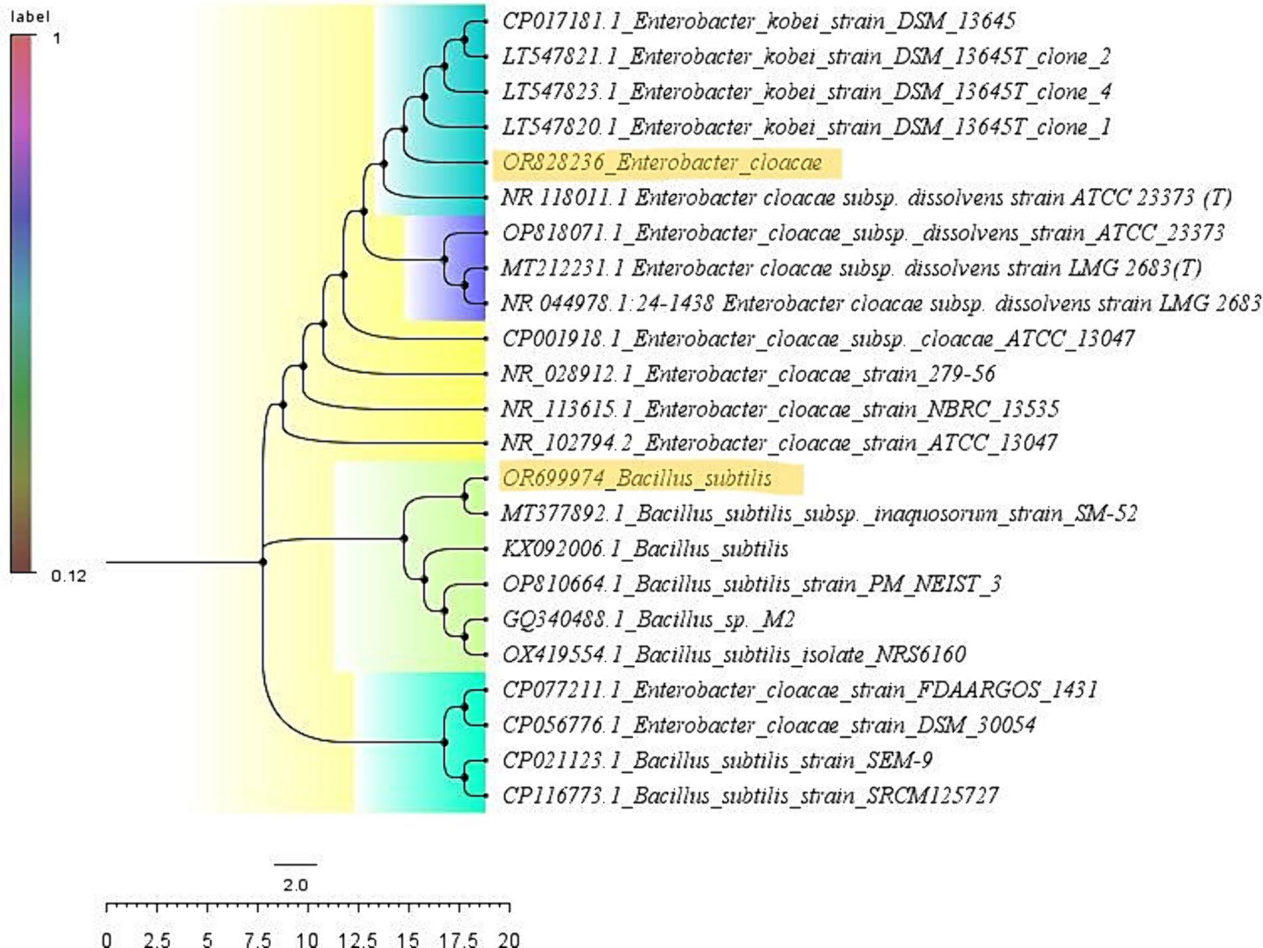
Phylogenetic tree depicting the analogy between the test isolates and type strains: the sequences were pairwise aligned with the type strains using the Clustal W method (with 1,000 bootstraps), and the resulting phylogenetic tree results were presented.

The rhizobacterial isolates PG-152 and PG-197 were subjected to molecular identification using 16SrDNA sequence analysis. Genomic DNA was isolated from both isolates. PCR-amplified DNA fragments of 1.3 kb products were analyzed for 16SrDNA sequencing. The sequences obtained from the Sanger sequence were blasted in NCBI-BLAST^1^ and BLAST tool in EzBioCloud^2^ for identifying the true identity of the isolates. The second tool was used to confirm the results obtained from the NCBI-BLAST. In the NCBI-BLAST, the *B. subtilis* sequence had an identity of 99.93% with a query cover of 99% with *B. subtilis* and *B. tequilensis* type strains (Table 3). In EzBioCloud, the same sequence had an identity of 99.72% with a query cover of 95.9% with *Bacillus tequilensis*. In the case of *Enterobacter* sp. sequence, when blasted in NCBI, it has a similarity of 98.81% with a query cover of 100% to *E. cloacae* type strains, whereas in EzBioCloud, it has 98.81% similarity with 51.8% coverage only. The nucleotide sequences were deposited in the NCBI GenBank, and the accession numbers obtained for the isolates PG-152 and PG-197 were OR828236 and OR699974, respectively. The phylogenetic tree was constructed using the Mega-XI software (Figure 5).

**Table 3:** Potential native PGPR isolates and their phylogenetic relationship.

Isolate code	Place	Habitat	Degree of similarity with nearest phylogenetic neighbor	GenBank accession number
PG-152	Vijayapura, Karnataka	Pigeon pea rhizosphere soil	*Bacillus subtilis* (99%)	OR699974
PG-197	Bagalkote, Karnataka	Sorghum rhizosphere soil	*Enterobacter* sp. (99%)	OR828236

Later, using the same sequences, mining was done for the open reading frames (ORF) embedded in the sequences using the open-source ORF founder.^3^ Parameters were selected with ATG or any other sense codon as a start codon, and the results were blasted in the protein BLAST. When *the Enterobacter* sp. sequence was used for the ORF finding, 26 ORFs were reported: 14 were positive strands, and 12 were negative strands. When blasted, most sequences were similar to the Enterobacteriaceae family’s hypothetical proteins. In the case of *B. subtilis*, ORF blast results had similarities with the 21 hypothetical proteins of Firmicutes (*Bacillus amyloliquefaciens, Streptococcus pneumoniae, B. subtilis, Lacticaseibacillus paracasei, Alkalihalophilus pseudofirmus, Rossellomorea aquimaris, Bacillus cereus, Pullulaniacillus purei, Staphylococcus aureus* (3), *Paenibacillus bouchesdurhonensis, and Coprobacillus* sp.), enterobacteria (*Klebsiella pneumoniae* and *Campylobacter jejuni*), high G + C bacteria (*Mycobacteroides abscessus*), fungus (*Glomus cerebriform*), and mayflies (*Ephemera danica*).

## Discussion

Due to topographical and forest demographic anomalies, northern Karnataka receives less rainfall, leading to poor crop yields and pressing the farmers economically. Though the area is water-enriched through dams and canal systems, the soil is plagued by salinity and alkalinity in the command areas. These abnormal environmental and edaphic conditions are escalating poverty and debriefing the sustainability of resources. Therefore, the people of northern Karnataka depend on climate–adaptive crops for their dietary and fodder requirements. The major crops include millets, and, to some extent, pulses were grown. The millets receive less or no fertilizer and water amendments throughout the crop growth period but still perform satisfactorily. It was long speculated that the crops were enriched with highly selective rhizosphere microflora attributed to their yield output. The microbial importance in the crop growth and yield of millets, especially sorghum, was largely unexplored and desperately needed at the moment. The selection and evaluation of test isolates assumed immense importance in agriculture and allied sectors for their potential applications. In light of microorganisms’ significant role in crop development and protection, the current study sought to identify potential growth-promoting microflora for the sorghum crop.

The rhizosphere is a hidden potential ecosystem for several beneficial microorganisms, which actively colonize the plant roots. They interact with plants, defend against soil-borne diseases, and play a crucial role in the soil nutrient pool’s transformation, mobilization, and solubilization, ensuring that plants balanced nutrition. Sorghum is a staple crop that is widely grown in semi-arid parts of the world, particularly in Africa and India. Since these rhizosphere microorganisms vary in terms of their existence and activities and have a variety of agricultural consequences, they are referred to as plant growth-promoting rhizobacteria (PGPR; Kloepper, 1978). Because of their great diversity, a thorough sampling was thus done in three districts in northern Karnataka. The preliminary selection of strains was performed based on the colony morphology, where different colony types were selected to maintain the diversity. They are inexpensive and environmentally benign and do not require non-renewable energy sources (Ahmad et al., 2008). The isolates are inherently present around the roots, but the number of isolates depends on the type of plant, soil conditions, and populations of other microbes. The scientific community commonly acknowledged and understood that replenishing the desired test isolate population in the rhizosphere is necessary to obtain the intended yields. The preliminary selection of strains was performed based on the colony variations, where different colony types were selected to maintain the diversity. These isolates improved seed germination by 3.5 times after inoculating the sorghum seeds. This improvement in germination was statistically significant (*p* < 0.01) and was attributed to increased water absorption. According to the current study, up to 85.5% of seeds can germinate when treated with *P. fluorescens*. Based on these test results, efficient cultures were allowed for further *in vitro* qualitative analysis.

Most rhizosphere-competent microorganisms have the critical ability to solubilize phosphorus, which is accomplished by generating organic acids or enzymes that begin interacting with the phosphorous minerals and salts in the soil (Brigido et al., 2017). The biosynthesis of organic acids is the key mechanism for solubilizing any insoluble mineral, including phosphorus (Nagaraju et al., 2017, 2020). Many studies contend that isolates processing phosphate solubilization-like properties are also capable of releasing zinc (Nagaraju et al., 2017), silicon, potassium (Flatian et al., 2021), and boron (Mandal et al., 2018) from minerals or complexes. On the other hand, the current study demonstrates that the isolates consistently showing strong phosphate solubilization were erratic in their potassium release. The test isolates’ phosphate solubilization complies with the requirements for commercial biofertilizers established by the Fertilizer Control Order (FCO) 1985, under Organic and Biological Inputs. The FCO 1985 standards recommend a minimum solubilization thickness of 5 mm in Pikovskaya’s medium layer of 3 mm for producing a phosphate-solubilizing biofertilizer. According to this investigation, the PG-197 test isolate had the greatest average phosphate solubilization, measuring 7.5 mm.

The test isolates *B. subtilis* (PG-152) could solubilize 2.69 g of inorganic phosphorous per ml of broth, according to the p solubilization broth assay results, which provide additional refinement of the evidence. The results were consistent with studies from various other countries. Li et al. (2021) studied phosphate solubilization by *B. subtilis* and *E. cloacae* in China and found that *E. cloacae* inoculation had higher solubilization than inoculation as a mixed culture. In addition to facilitating the biogeochemical cycling of nutrients, the rhizosphere isolates have been demonstrated to be able to produce chemicals that support plant growth (Priyanka et al., 2017; Geeta et al., 2018; Harshitha and Goudar, 2021). Leite et al. (2013) examined the ability of endophytic bacterial cultures from Bahia to promote the healthy development of cocoa, including *Enterobacter cloacae* and *B. subtilis*. The rationale was designated to the biosynthesis of phytohormones such as cytokinins, auxins, and ethylene. It was discovered that early colonization by *Enterobacter* sp. and *B. subtilis* improved the root architecture and produced indole acetic acid (Ansuman Senapati et al., 2021). The auxins that rhizobacteria produce support the growth of the root system, make it easier to absorb nutrients, and encourage more biomass formation (Vikram, 2007). Indole acetic acid and gibberellic acid are the two main phytohormones that affect plant growth. It has been shown that various rhizosphere bacteria create phytohormones by interfering with biosynthetic routes or by using inbuilt biosynthetic pathways. The production of IAA by bacteria uses tryptophan as a precursor. In three steps, the indole-3-pyruvate route (IPyA) in *Enterobacter cloacae* produces indole acetic acid (Goudar, 2021).

Furthermore, Kalam et al. (2020) isolated seven bacteria from the tomato rhizosphere that were known to have plant growth-promoting (PGP) characteristics, such as the ability to solubilize phosphate and zinc, and produce hydrogen cyanide (HCN), IAA, phytase, siderophore, and ACC deaminase. The seven isolates were recognized as *Bacillus* sp. by examining the 16S rDNA sequence. The solubilization effectiveness of isolates/strains is influenced by the soil’s physiochemical characteristics (Alori et al., 2017; Maaloum et al., 2020), strain population (Bagyalakshmi et al., 2017), plant and microbial physiology (Chen et al., 2021), soil nutrient status, and the niche’s current ecology (Liang et al., 2020). Our findings suggest that most isolates possess multiple PGPR activities rather than a single character. In addition, most native isolates may be significantly more hostile than commercial bioinoculants, necessitating thorough screening for prospective strains. The syntheses of siderophore and HCN, which cause systemic resistance, are traits of certain isolates that make them effective biocontrol agents. Numerous studies have shown how the generation of HCN, siderophores, and antibiotics by Pseudomonas strains inhibits plant diseases such as *F. oxysporum* and *R. solani* (Stijn et al., 2007; Ahmad et al., 2008). *Pseudomonas fluorescens* produce numerous antibiotics with various effects against certain pathogenic fungi strains CHA0 and Pf5 (Raaijmakers and Weller, 1998). Rhizobacteria can biocontrol a variety of fungal phytopathogens, according to Ali et al. (2020). The significance of *B. subtilis* as a possible biocontrol agent against *F. oxysporum* and *M. phaseolina* was emphasized by Singh et al. (2008). These fungi impede mycelial development by producing chitinase and − 1,3-glucanase, which results in the rupture of the fungal cell wall (Skujins et al., 1965).

This study found a significant coefficient of variation (CV) for measures of the shoot length, root length, and seed vigor index for the PGPR-inoculated plants. When tomato seedlings were bacterized with the top five rhizosphere isolates, Qessaoui et al. (2019) discovered that the germination percentage was increased many times. This may be linked to the type IV pili of Pseudomonad, which regulates the balance of seed hormones, such as gibberellic acid (GA) and indole acetic acid (IAA), and aids adherence to the host plant (Bharathi et al., 2004). Significantly, the lowest germination rate (51.0%) was reported in plants treated with the PG-114 strain. A recent study on Arabidopsis plants demonstrated seeds’ likelihood of germinating less frequently when other living things are present. Pseudomonad species, including *P. aeruginosa*, are well recognized for limiting seed germination in Arabidopsis by producing the chemical L-2-amino-4-methoxy-trans-3-butenoic acid (AMB), whose synthesis is governed by quorum sensing (Chahtane et al., 2018).

The PGPR has been linked to synthesizing phytohormones and other direct modes of action for promoting plant development. While *B. cereus* generates IAA (Nagaraju et al., 2021), solubilizes phosphate (Lifshitz et al., 1986), and also makes ACC deaminase, which aids in reducing ethylene concentration (Glick, 1995; Nagaraju et al., 2021), it is never easy to determine whether PGPR supports plant development by employing only one mechanism of action. The results obtained with isolate PG-152 demonstrate the formation of siderophores, phosphate, Zn solubilization, and IAA and GA. The experiment did not subject the plants to iron restriction; hence, the impact of siderophores on promoting plant development was not considered. The other methods, individually or in combination, may be responsible for the isolate’s ability to promote sorghum development. The biosynthesis of IAA by rhizobacteria may mediate response to light and gravity, impacting photosynthesis via increased chlorophyll and biosynthesis of different metabolites and resilience to stress conditions. IAA and GA lengthen roots, increase plant growth, and lengthen shoots (Wang et al., 2012).

At 30, 60, and 90 DAS, plants inoculated with the reference strain (*P. fluorescens*) and PG-152 (*B. subtilis*) recorded their maximum shoot dry weight, dry root weight, and total dry weight. Competent isolates that produce growth hormones such as IAA, gibberellins, and auxins were sought to be the root cause of the increase (Li et al., 2021). The auxin generated by rhizobacteria can enhance the root system’s growth and the absorption of vital nutrients for plant growth, which increases the amount of biomass produced (Vikram, 2007). PGPR-inoculated maize had a much higher biomass than the control (Agbodjato et al., 2015). The plants inoculated with a mixture of *P. fluorescens* and *P. putida* showed the largest increases in shoot and root biomass, respectively, at 53.72 and 108.71% (Gholami et al., 2009). Similar findings were made by Dobbelaere et al. (2001), who claimed that wheat plants inoculated with *Azospirillum brasilense* enhanced the dry weight of the root and shoot systems.

Rhizobacteria inoculation improved the yield of sorghum in comparison with the uninoculated control. The reference strain (*P. fluorescens*) recorded the highest grain yield of 41.62 g/plant, followed by the PG-152 (36.13 g/plant). The same plants also showed proportional nitrogen and phosphorus absorption, indicating that these strains supply the nutrients that increased production. According to several pieces of research, PGPR improves growth, seed emergence, and crop production (Dey et al., 2004; Kloepper et al., 2004). According to Agbodjato et al. (2015), PGPR-treated maize increased its dry weight the most compared to uninoculated treatment. In plants inoculated with a mixture of *P. fluorescens* and *P. putida*, the shoot and root biomass increased by the greatest percentages of 53.72 and 108.71%, respectively (Gholami et al., 2009). Similarly, Dobbelaere et al. (2001) noted that wheat plants inoculated with *A. brasilense* had enhanced the dry weight of the root system and overall dry weight.

## Conclusion

Globally, chemical and pesticide use has become common in modern agricultural practices, seriously impacting the soil and ecology. A deep knowledge gap between the microbial role in sustaining the resources and their deep roots in the ecological balance has been created due to the preferential bias. In the current study, the rhizosphere isolates were found to enhance the germination capacity of the sorghum seeds. They were also found to inhibit the pathogen attack, such as *Macrophomina* sp. and *Fusarium* sp., and enhance the nitrogen and phosphorous nutrient uptake. In the pot culture experiment, the isolates increased plant height, chlorophyll content, and biomass accumulation over a period. The isolate’s identity was revealed using biochemical, morphological, and molecular techniques. However, there is a lot of scope to understand the inoculant’s potential through field evaluation, assuming their immense importance in agriculture and biotechnology as a commercial inoculant.

## Data availability statement

The datasets presented in this study can be found in online repositories. The names of the repository/repositories and accession number(s) can be found at: https://www.ncbi.nlm.nih.gov/, OR699974<br>https://www.ncbi.nlm.nih.gov/, OR828236.

## Author contributions

MC: Conceptualization, Investigation, Methodology, Writing – review & editing. GG: Conceptualization, Methodology, Resources, Supervision, Writing – review & editing. KP: Conceptualization, Investigation, Methodology, Writing – review & editing, Formal analysis, Funding acquisition, Supervision, Validation. NY: Data curation, Formal analysis, Investigation, Methodology, Project administration, Resources, Software, Supervision, Validation, Visualization, Writing – original draft, Writing – review & editing.

## Footnotes

^1^https://blast.ncbi.nlm.nih.gov/Blast.cgi?PROGRAM=blastn&PAGE_TYPE=BlastSearch&LINK_LOC=blasthome

^2^https://www.ezbiocloud.net/identify

^3^https://www.ncbi.nlm.nih.gov/orffinder/
