# Supplementary material for: Study on effect of plant growth promoting rhizobacteria on sorghum (Sorghum bicolor L.) under gnotobiotic conditions
Source: Front Microbiol. 2024 Oct 4;15:1374802. doi: 10.3389/fmicb.2024.1374802 (PMC11529481; doi:10.3389/fmicb.2024.1374802)
Supplement: Supplementary file 1 [file Table_1.DOCX]

**Title**

**The functional characterization and microcosm evaluation of sorghum plant invigorating rhizobacteria**

M. Chiranjeevi^1^, G. D. Goudar^2^ and Yalavarthi Nagaraju^3^*

^1^Department of Agricultural Microbiology, College of Agriculture, Vijayapur-586101

^2^Department of Agricultural Microbiology, College of Agriculture, Dharwad-580005

^3^ICAR-NBAIM, Kushmaur, Uttar Pradesh-275103

Journal Name: current Microbiology

*Corresponding author

[nagarajulvrth62@gmail.com](mailto:nagarajulvrth62@gmail.com)

**Supplementary Table 1: Details of sampling site and samples collected for the isolation of PGPR**

| **1a. Vijayapura District** | | | | | | | | | |
| --- | --- | --- | --- | --- | --- | --- | --- | --- | --- |
| **Sl.**  **No.** | **Place** | **Source** | **Soil type** | **Crop** | **GPS values** | | | **Number of isolates** | **code of the isolates** |
|  |  |  |  |  | **Latitude (N)** | **Longitude (E)** | **Elevation**  **(m)** |  |  |
| 1 | Kannur | Rhizosphere soil | Black soil | Maize | 17^0^ 02' 960" | 75^0^ 41' 946" | 577 | 4 | PG |
| 2 | Kannur | Rhizosphere soil | Black soil | Chickpea | 17^0^ 03' 070" | 75^0^ 41' 961" | 578 | 5 | PG |
| 3 | Mahaveer nagar | Rhizosphere soil | Red soil | Tomato | 17^0^ 01'087" | 75^0^ 14' 048" | 581 | 6 | PG |
| 4 | Mahaveer nagar | Rhizosphere soil | Black soil | Pigeon pea | 17^0^ 08' 090" | 75^0^ 21' 896" | 588 | 5 | PG |
| 5 | Mahaveer nagar | Rhizosphere soil | Black soil | Sorghum | 17^0^ 26' 010" | 75^0^ 23' 594" | 592 | 4 | PG |
| 6 | Inchageri | Rhizosphere soil | - | Pigeon pea | 17^0^ 0' 978" | 75^0^ 23' 876" | 532 | 6 | PG |
| 7 | Inchageri | Rhizosphere soil | Red soil | Pigeon pea | 17^0^ 25' 978" | 75^0^ 23' 874" | 538 | 5 | PG |
| 8 | Inchageri | Rhizosphere soil | Red soil | Sorghum | 17^0^ 26' 921" | 75^0^ 28' 462" | 535 | 5 | PG |
| 9 | Inchageri | Rhizosphere soil | - | Banana | 17^0^ 26' 777" | 75^0^ 29' 211" | 546 | 4 | PG |
| 10 | Lamana tanda | Rhizosphere soil | Black soil | Pigeon pea | 17^0^ 26' 775" | 75^0^ 29' 211" | 557 | 5 | PG |
| 11 | Lamana tanda | Rhizosphere soil | Black soil | Chickpea | 17^0^ 32' 399" | 75^0^ 38' 183" | 559 | 4 | PG |
| 12 | Lamana tanda | Rhizosphere soil | - | Sugarcane | 17^0^ 27' 398" | 75^0^ 38' 105" | 562 | 5 | PG |
| 13 | Lamana tanda | Rhizosphere soil | Black soil | Maize | 17^0^ 33' 393" | 75^0^ 34' 289" | 568 | 4 | PG |
| 14 | Jigaievani | Rhizosphere soil | Black soil | Maize | 17^0^ 44' 395" | 75^0^ 37' 191" | 575 | 3 | PG |
| 15 | Jigaievani | Rhizosphere soil | Black soil | Pigeon pea | 17^0^ 27' 647" | 75^0^ 34' 600" | 579 | 6 | PG |
| 16 | Katral | Rhizosphere soil | Black soil | Pigeon pea | 17^0^ 32' 398" | 75^0^ 38' 108" | 495 | 5 | PG |
| 17 | Lamana hatti | Rhizosphere soil | Black soil | Pigeon pea | 17^0^ 44' 393" | 75^0^ 44' 926" | 514 | 5 | PG |
| 18 | Hadalsang | Rhizosphere soil | - | Grape | 17^0^ 53' 108" | 75^0^ 50' 467" | 504 | 4 | PG |
| 19 | Hadalsang | Rhizosphere soil | Black soil | Sorghum | 17^0^ 54' 040" | 75^0^ 51' 901" | 508 | 5 | PG |
| 20 | Hadalsang | Rhizosphere soil | Black soil | Pigeon pea | 17^0^ 54' 040" | 75^0^ 51' 901" | 519 | 3 | PG |
| 21 | Hadalsang | Rhizosphere soil | Black soil | Pigeon pea | 17^0^ 54' 040" | 75^0^ 51' 901" | 511 | 4 | PG |
| 22 | Hadalsang | Rhizosphere soil | Black soil | Sorghum | 17^0^ 54' 040" | 75^0^ 51' 901" | 518 | 2 | PG |

| **1B. Kalaburagi District** | | | | | | | | | |
| --- | --- | --- | --- | --- | --- | --- | --- | --- | --- |
| **Sl.**  **No.** | **Place** | **Source** | **Soil type** | **Crop** | **GPS values** | | | **Number of isolates** | **Code of the isolates** |
|  |  |  |  |  | **Latitude (N)** | **Longitude (E)** | **Elevation**  **(m)** |  |  |
| 1 | Kalagi | Rhizosphere soil | Black soil | Maize | 17^0^ 24' 467" | 76^0^ 85' 258" | 458 | 4 | PG |
| 2 | Karajagi | Rhizosphere soil | Red soil | Sorghum | 17^0^ 33' 362" | 76^0^ 25' 202" | 461 | 5 | PG |
| 3 | Karajagi | Rhizosphere soil | Red soil | Sunflower | 17^0^ 30'777" | 76^0^ 25' 143" | 446 | 5 | PG |
| 4 | Karajagi | Rhizosphere soil | Red soil | Pigeon pea | 17^0^ 30' 324" | 76^0^ 26' 938" | 436 | 5 | PG |
| 5 | Kalagi | Rhizosphere soil | Black soil | Pigeon pea | 17^0^35' 555" | 77^0^ 15' 886" | 482 | 6 | PG |
| 6 | Alagudda | Rhizosphere soil | Black soil | Sorghum | 17^0^ 43' 736" | 76^0^ 91' 575" | 483 | 3 | PG |
| 7 | Alagudda | Rhizosphere soil | Black soil | Pigeon pea | 17^0^ 43' 748" | 76^0^ 91' 613" | 487 | 4 | PG |
| 8 | Alagudda | Rhizosphere soil | Black soil | Sorghum | 17^0^ '43 "753 | 76^0^ '91 "643 | 491 | 5 | PG |
| 9 | Dharmapura | Rhizosphere soil | Black soil | cotton | 17^0^ '23 "088 | 76^0^ 90' "261 | 432 | 4 | PG |
| 10 | Shankerwadi | Rhizosphere soil | Black soil | Pigeon pea | 17^0^ '14 "077 | 76^0^ '95 "445 | 433 | 5 | PG |
| 11 | Nalvar | Rhizosphere soil | Red soil | Sorghum | 16^0^ 95' 045" | 77^0^ 07' 627" | 399 | 4 | PG |
| 12 | Kollur | Rhizosphere soil | Red soil | Sorghum | 16^0^ 94' 382" | 77^0^ 01' 091" | 412 | 5 | PG |
| 13 | Mudhol | Rhizosphere soil | Black soil | Maize | 16^0^ 92' 939" | 77^0^ 44' 784" | 589 | 4 | PG |
| 14 | Bheemanalli | Rhizosphere soil | Black soil | Maize | 16^0^ 95' 38" | 77^0^ 21' 367" | 513 | 6 | PG |
| 15 | Vidyagiri | Rhizosphere soil | Black soil | Pigeon pea | 16^0^17'863 " | 75^0^ 65' 752" | 538 | 5 | PG |

| **1c. Bagalkot Diistrict** | | | | | | | | | |
| --- | --- | --- | --- | --- | --- | --- | --- | --- | --- |
| **Sl.**  **No.** | **Place** | **Source** | **Soil type** | **Crop** | **GPS values** | | | **Number of isolates** | **code of the isolates** |
|  |  |  |  |  | **Latitude (N)** | **Longitude (E)** | **Elevation**  **(m)** |  |  |
| 1 | Sokanadagi | Rhizosphere soil | Black soil | Maize | 16^023^ '409 " | 75^0^ 56' 645" | 528 | 3 | PG |
| 2 | Kovalli | Rhizosphere soil | Red soil | Sorghum | 16^0^ 23' 808" | 75^0^ 58' 536" | 526 | 2 | PG |
| 3 | Budihala | Rhizosphere soil | Red soil | Sunflower | 16^0^ 24'273" | 75^0^ 59' 186" | 528 | 1 | PG |
| 4 | Bavalatti | Rhizosphere soil | Red soil | Pigeon pea | 16^0^ 24' 962" | 75^0^ 57' 113" | 533 | 3 | PG |
| 5 | Siddapura | Rhizosphere soil | Black soil | Pigeon pea | 16^0^37' 995" | 75^0^ 55' 288" | 538 | 2 | PG |
| 6 | Simikeri | Rhizosphere soil | Black soil | Pigeon pea | 16^0^ 17'696" | 75^0^ 61'050" | 541 | 3 | PG |
| 7 | Keruru | Rhizosphere soil | Red soil | Pigeon pea | 16^0^ 01' 150" | 75^0^ 54' 637" | 638 | 3 | PG |
| 8 | Jalageri | Rhizosphere soil | Red soil | Grape | 16^0^ 10'449" | 75^0^ 54' 535" | 582 | 2 | PG |
| 9 | Kagaagumba | Rhizosphere soil | Red soil | Sorghum | 16^0^ 11' 010" | 75^0^ 62' 069" | 583 | 3 | PG |
| 10 | Yadahalli | Rhizosphere soil | Black soil | Pigeon pea | 16^0^ 21' 675" | 75^0^61' 269" | 528 | 2 | PG |
| 11 | Kontikalla | Rhizosphere soil | Black soil | Sorghum | 16^0^37' 903" | 75^0^ 58' 841" | 535 | 3 | PG |
| 12 | Kontikalla | Rhizosphere soil | Black soil | Pigeon pea | 16^0^37' 903" | 75^0^ 58' 841" | 532 | 2 | PG |
| 13 | Kontikalla | Rhizosphere soil | Black soil | Pigeon pea | 16^0^37' 903" | 75^0^ 58' 841" | 534 | 1 | pg |

**Supplementary Table 2: Rapid screening of the native rhizobacterial isolates for plant growth promotion activities by roll towel method**.

| **Sl.No** | **Isolates** | **Mean shoot length** | **Mean root length** | **Germination percentage** | **Vigour index-I** |
| --- | --- | --- | --- | --- | --- |
| 1 | PG-1 | 8.70 | 5.45 | 72.00 (49.89)* | 1018.55 |
| 2 | PG-2 | 9.85 | 5.52 | 67.50 (58.05) | 1037.80 |
| 3 | PG-3 | 8.51 | 6.06 | 75.50 (60.33) | 1099.92 |
| 4 | PG-4 | 9.25 | 6.38 | 73.50 (55.24) | 1149.23 |
| 5 | PG-5 | 10.45 | 7.25 | 69.00 (52.53) | 1221.50 |
| 6 | PG-6 | 10.25 | 7.35 | 69.00 (52.53) | 1214..40 |
| 7 | PG-7 | 9.80 | 5.30 | 69.50 (56.47) | 1049.45 |
| 8 | PG-8 | 10.25 | 4.37 | 62.50 (51.64) | 0914.02 |
| 9 | PG-9 | 11.40 | 6.62 | 61.50 (51.84) | 1107.97 |
| 10 | PG-10 | 10.25 | 6.56 | 52.50 (46.43) | 0882.81 |
| 11 | PG-11 | 9.40 | 6.25 | 63.50 (52.83) | 0994.75 |
| 12 | PG-12 | 8.36 | 4.25 | 65.50 (54.02) | 826.86 |
| 13 | PG-13 | 9.40 | 5.55 | 69.50 (56.47) | 1039.10 |
| 14 | PG-14 | 10.25 | 6.30 | 63.50 (52.83) | 1051.45 |
| 15 | PG-15 | 9.10 | 8.25 | 64.50 (53.42) | 1119.30 |
| 16 | PG-16 | 8.75 | 5.70 | 67.50 (55.24) | 0975.30 |
| 17 | PG-17 | 7.70 | 5.45 | 53.50 (47.00) | 0704.05 |
| 18 | PG-18 | 8.70 | 6.44 | 72.00 (58.05) | 1090.42 |
| 19 | PG-19 | 8.50 | 5.39 | 72.50 (58.37) | 1007.08 |
| 20 | PG-20 | 6.70 | 6.35 | 74.00 (49.81) | 0965.65 |
| 21 | PG-21 | 8.25 | 5.60 | 63.50 (44.89) | 0879.25 |
| 22 | PG-22 | 8.40 | 5.35 | 61.50 (42.07) | 0845.60 |
| 23 | PG-23 | 10.35 | 6.10 | 77.50 (61.68) | 1274.75 |
| 24 | PG-24 | 10.25 | 7.70 | 74.50 (59.67) | 1337.05 |
| 25 | PG-25 | 9.70 | 7.30 | 61.00 (51.35) | 1036.70 |
| 26 | PG-26 | 8.15 | 6.22 | 73.50 (59.07) | 1056.55 |
| 27 | PG-27 | 9.35 | 5.61 | 60.50 (51.06) | 0905.29 |
| 28 | PG-28 | 8.55 | 7.15 | 75.00 (60.00) | 1177.70 |
| 29 | PG-29 | 8.15 | 5.45 | 71.00 (57.41) | 0965.70 |
| 30 | PG-30 | 9.35 | 7.35 | 63.00 (52.53) | 1051.80 |
| 31 | PG-31 | 7.55 | 7.40 | 65.00 (53.72) | 0972.20 |
| 32 | PG-32 | 8.55 | 7.27 | 64.00 (53.17) | 1012.85 |
| 33 | PG-33 | 7.15 | 6.45 | 75.00 (60.00) | 1020.00 |
| 34 | PG-34 | 6.82 | 5.25 | 75.00 (60.00) | 905.25 |
| 35 | PG-35 | 7.35 | 6.05 | 65.00 (53.72) | 0870.90 |
| 36 | PG-36 | 8.25 | 5.25 | 62.00 (51.94) | 0837.00 |
| 37 | PG-37 | 9.70 | 6.70 | 56.00 (48.44) | 0918.80 |
| 38 | PG-38 | 9.45 | 6.55 | 73.00 (58.69) | 1168.00 |
| 39 | PG-39 | 5.70 | 5.70 | 72.00 (58.05) | 0820.40 |
| 40 | PG-40 | 8.40 | 6.40 | 70.00 (56.78) | 1036.00 |
| 41 | PG-41 | 7.70 | 7.10 | 79.50 (63.07) | 1176.55 |
| 42 | PG-42 | 8.25 | 6.85 | 68.00 (55.55) | 1026.50 |
| 43 | PG-43 | 7.35 | 7.35 | 77.00 (61.34) | 1147.30 |
| 44 | PG-44 | 8.35 | 7.45 | 79.50 (63.07) | 1256.10 |
| 45 | PG-45 | 8.60 | 7.85 | 78.00 (62.02) | 1283.40 |
| 46 | PG-46 | 7.45 | 6.55 | 67.00 (54.07) | 0938.15 |
| 47 | PG-47 | 8.57 | 5.40 | 79.00 (63.07) | 1103.63 |
| 48 | PG-48 | 8.30 | 4.94 | 71.00 (57.41) | 0939.78 |
| 49 | PG-49 | 7.30 | 5.70 | 72.0 (58.05) | 0936.20 |
| 50 | PG-50 | 7.70 | 6.40 | 73.0 (58.69) | 1027.50 |
| 51 | PG-51 | 8.50 | 7.35 | 74.0 ( 59.34) | 1172.70 |
| 52 | PG-52 | 9.40 | 7.20 | 74.0 (59.34) | 1228.60 |
| 53 | PG-53 | 8.25 | 7.23 | 76.0 (60.66) | 1176.03 |
| 54 | PG-54 | 5.45 | 4.45 | 59.5 (50.47) | 0588.65 |
| 55 | PG-55 | 10.13 | 7.20 | 76.0 (60.66) | 1316.33 |
| 56 | PG-56 | 9.85 | 6.45 | 70.0 (56.78) | 1140.90 |
| 57 | PG-57 | 9.40 | 5.30 | 63.0 (52.53) | 0889.40 |
| 58 | PG-58 | 8.65 | 6.50 | 77.0 (61.34) | 1167.25 |
| 59 | PG-59 | 10.13 | 5.45 | 74.0 (59.34) | 1151.60 |
| 60 | PG-60 | 9.95 | 4.13 | 64.0 (53.13) | 0900.73 |
| 61 | PG-61 | 10.85 | 5.40 | 66.5 (54.63) | 1080.55 |
| 62 | PG-62 | 7.35 | 5.35 | 74.0 (59.34) | 0940.40 |
| 63 | PG-63 | 10.40 | 5.60 | 71.5 (57.73) | 1144.90 |
| 64 | PG-64 | 10.10 | 5.20 | 70.5 (57.10) | 1078.50 |
| 65 | PG-65 | 7.65 | 5.60 | 74.5 (59.67) | 0987.12 |
| 66 | PG-66 | 9.20 | 5.35 | 72.5 (58.05) | 1054.87 |
| 67 | PG-67 | 10.35 | 7.40 | 70.5 (57.10) | 1251.40 |
| 68 | PG-68 | 8.30 | 7.13 | 74.5 (59.67) | 1149.08 |
| 69 | PG-69 | 10.40 | 7.75 | 72.0 (58.05) | 1307.16 |
| 70 | PG-70 | 10.10 | 5.90 | 70.5 (57.10) | 1128.05 |
| 71 | PG-71 | 10.45 | 6.69 | 65.5 (54.02) | 1122.57 |
| 72 | PG-72 | 9.95 | 6.12 | 72.5 (58.37) | 1164.63 |
| 73 | PG-73 | 7.35 | 6.85 | 64.0 (53.13) | 0908.80 |
| 74 | PG-74 | 7.55 | 5.55 | 75.5 (60.33) | 0988.90 |
| 75 | PG-75 | 7.40 | 7.43 | 72.5 (58.37) | 1074.89 |
| 76 | PG-76 | 8.15 | 7.20 | 76.5 (61.02) | 1174.05 |
| 77 | PG-77 | 9.35 | 8.18 | 76.0 (60.66) | 1332.71 |
| 78 | PG-78 | 10.00 | 7.28 | 72.5 (58.37) | 1252.40 |
| 79 | PG-79 | 11.45 | 7.65 | 74.5 (59.67) | 1422.95 |
| 80 | PG-80 | 10.10 | 6.25 | 74.5 (59.67) | 1218.30 |
| 81 | PG-81 | 8.05 | 5.85 | 72.5 (58.37) | 1008.95 |
| 82 | PG-82 | 7.20 | 6.20 | 74.5 (59.67) | 0998.45 |
| 83 | PG-83 | 7.20 | 6.35 | 75.5 (60.33) | 1022.95 |
| 84 | PG-84 | 10.30 | 6.70 | 77.0 (61.34) | 1308.80 |
| 85 | PG-85 | 10.12 | 6.40 | 73.0 (58.69) | 1205.34 |
| 86 | PG-86 | 9.60 | 6.35 | 71.5 (57.73) | 1140.05 |
| 87 | PG-87 | 8.12 | 5.33 | 77.0 (64.89) | 1035.65 |
| 88 | PG-88 | 7.60 | 4.60 | 71.5 (57.37) | 0872.30 |
| 89 | PG-89 | 8.85 | 5.70 | 66.5 (54.63) | 0967.45 |
| 90 | PG-90 | 11.35 | 7.30 | 71.5 (57.73) | 1333.25 |
| 91 | PG-91 | 9.65 | 5.25 | 78.0 (62.02) | 1162.10 |
| 92 | PG-92 | 11.01 | 6.85 | 77.5 (61.68) | 1384.13 |
| 93 | PG-93 | 10.10 | 7.18 | 77.0 (61.34) | 1330.53 |
| 94 | PG-94 | 8.75 | 6.50 | 79.5 (63.07) | 1212.30 |
| 95 | PG-95 | 9.65 | 5.30 | 69.5 (56.47) | 1039.15 |
| 96 | PG-96 | 11.10 | 6.85 | 76.0(60.66) | 1364.35 |
| 97 | PG-97 | 9.15 | 5.55 | 74.5(59.67) | 1095.45 |
| 98 | PG-98 | 9.05 | 5.55 | 74.5(59.67) | 1087.57 |
| 99 | PG-99 | 11.50 | 7.20 | 69.5 (56.47) | 1299.58 |
| 100 | PG-100 | 10.65 | 6.19 | 69.0 (56.16) | 1161.75 |
| 101 | PG-101 | 9.55 | 6.10 | 70.0 (56.78) | 1095.50 |
| 102 | PG-102 | 7.55 | 5.30 | 70.0 (56.78) | 0899.50 |
| 103 | PG-103 | 10.41 | 6.50 | 54.0 (47.29) | 0915.58 |
| 104 | PG-104 | 7.17 | 6.73 | 61.0 (51.35) | 0848.16 |
| 105 | PG-105 | 10.74 | 6.25 | 52.5 (46.43) | 0890.75 |
| 106 | PG-106 | 11.45 | 6.35 | 54.5 (47.58) | 0969.10 |
| 107 | PG-107 | 12.10 | 7.03 | 72.0 (58.05) | 1377.25 |
| 108 | PG-108 | 9.45 | 7.30 | 73.0 (58.69) | 1223.10 |
| 109 | PG-109 | 8.75 | 7.13 | 77.0 (61.34) | 1222.35 |
| 110 | PG-110 | 9.20 | 6.58 | 62.0 (51.94) | 0978.30 |
| 111 | PG-111 | 7.65 | 6.70 | 71.0 (57.41) | 1018.40 |
| 112 | PG-112 | 6.70 | 6.10 | 53.0 (46.71) | 0677.50 |
| 113 | PG-113 | 6.74 | 6.55 | 54.0 (47.29) | 0717.66 |
| 114 | PG-114 | 6.38 | 5.15 | 51.0 (45.57) | 0587.55 |
| 115 | PG-115 | 10.30 | 5.25 | 71.0 (57.41) | 1103.80 |
| 116 | PG-116 | 9.50 | 7.45 | 62.0 (51.94) | 1050.40 |
| 117 | PG-117 | 10.70 | 5.40 | 63.0 (52.53) | 1014.30 |
| 118 | PG-118 | 10.35 | 5.13 | 71.0 (57.41) | 1098.70 |
| 119 | PG-119 | 8.60 | 7.25 | 53.0 (46.71) | 0839.60 |
| 120 | PG-120 | 11.10 | 6.45 | 62.5 (52.23) | 1098.00 |
| 121 | PG-121 | 7.85 | 6.58 | 73.0 (58.69) | 1053.30 |
| 122 | PG-122 | 6.70 | 7.05 | 70.5 (57.10) | 0969.55 |
| 123 | PG-123 | 7.60 | 5.43 | 62.5 (52.23) | 0814.30 |
| 124 | PG-124 | 7.50 | 5.13 | 70.5 (57.10) | 0890.28 |
| 125 | PG-125 | 7.75 | 6.60 | 61.0 (51.35) | 0875.30 |
| 126 | PG-126 | 10.25 | 7.70 | 64.0 (53.13) | 1148.55 |
| 127 | PG-127 | 7.45 | 5.29 | 68.5 (55.85) | 0872.18 |
| 128 | PG-128 | 7.90 | 6.60 | 79.5 (63.07) | 1152.70 |
| 129 | PG-129 | 9.80 | 7.40 | 77.0 (61.34) | 1324.00 |
| 130 | PG-130 | 10.75 | 6.45 | 52.5 (46.43) | 0903.5 |
| 131 | PG-131 | 7.65 | 7.30 | 62.5 (52.23) | 0933.5 |
| 132 | PG-132 | 9.70 | 7.67 | 70.5 (57.10) | 1224.55 |
| 133 | PG-133 | 10.40 | 6.25 | 75.5 (60.33) | 1257.25 |
| 134 | PG-134 | 11.75 | 7.70 | 71.5 (57.73) | 1390.85 |
| 135 | PG-135 | 9.45 | 7.19 | 77.0 (61.34) | 1281.56 |
| 136 | PG-136 | 11.35 | 7.10 | 60.5 (51.06) | 1116.45 |
| 137 | PG-137 | 6.20 | 5.14 | 54.5 (47.58) | 0617.76 |
| 138 | PG-138 | 9.55 | 7.25 | 63.0 (52.23) | 1059.6 |
| 139 | PG-139 | 9.15 | 5.30 | 73.0 (58.69) | 1054.95 |
| 140 | PG-140 | 7.10 | 4.65 | 59.5 (50.47) | 0699.25 |
| 141 | PG-141 | 6.70 | 7.85 | 64.5 (53.42) | 0918.45 |
| 142 | PG-142 | 9.04 | 7.57 | 60.5 (51.06) | 1004.72 |
| 143 | PG-143 | 8.45 | 6.52 | 55.5 (48.15) | 0830.32 |
| 144 | PG-144 | 10.60 | 6.10 | 62.0 (51.94) | 1036.20 |
| 145 | PG-145 | 10.25 | 7.95 | 82.5(65.27) | 1500.12 |
| 146 | PG-146 | 8.25 | 7.30 | 71.0 (57.41) | 1104.20 |
| 147 | PG-147 | 9.30 | 7.55 | 75.5 (60.33) | 1272.30 |
| 148 | PG-148 | 11.45 | 8.14 | 80.0 (66.42) | 1567.20 |
| 149 | PG-149 | 9.95 | 7.70 | 62.0 (51.94) | 1094.60 |
| 150 | PG-150 | 9.40 | 5.30 | 70.5 (57.10) | 1036.00 |
| 151 | PG-151 | 7.30 | 7.70 | 74.5 (59.67) | 1117.30 |
| 152 | PG-152 | 13.00 | 8.50 | 82.5 (65.27) | 1774.01 |
| 153 | PG-153 | 9.45 | 7.70 | 66.5 (54.63) | 1140.35 |
| 154 | PG-154 | 9.45 | 7.30 | 68.0 (55.55) | 1139.15 |
| 155 | PG-155 | 9.13 | 7.19 | 69.0 (56.16) | 1126.29 |
| 156 | PG-156 | 9.20 | 7.10 | 58.5 (49.89) | 1011.45 |
| 157 | PG-157 | 10.30 | 7.70 | 62.5 (52.23) | 1149.00 |
| 158 | PG-158 | 9.50 | 7.75 | 52.5 (46.43) | 0905.25 |
| 159 | PG-159 | 7.05 | 7.20 | 59.5 (50.47) | 0847.90 |
| 160 | PG-160 | 9.10 | 7.30 | 67.0 (54.93) | 1098.40 |
| 161 | PG-161 | 7.30 | 8.10 | 64.5 (53.42) | 0994.00 |
| 162 | PG-162 | 8.45 | 7.45 | 59.0 (50.18) | 0938.70 |
| 163 | PG-163 | 6.77 | 5.90 | 63.0 (52.53) | 0772.95 |
| 164 | PG-164 | 8.05 | 7.35 | 57.5 (49.31) | 0885.50 |
| 165 | PG-165 | 10.45 | 7.85 | 78.5 (63.43) | 1436.50 |
| 166 | PG-166 | 8.60 | 6.85 | 73.0 (58.69) | 1128.75 |
| 167 | PG-167 | 7.70 | 4.60 | 59.5 (50.47) | 0732.00 |
| 168 | PG-168 | 7.55 | 4.95 | 70.5 (57.10) | 0881.40 |
| 169 | PG-169 | 7.75 | 5.35 | 77.0 (61.34) | 1008.50 |
| 170 | PG-170 | 6.75 | 4.80 | 59.5 (50.18) | 0687.22 |
| 171 | PG-171 | 9.85 | 6.18 | 59.0 (60.00) | 0946.00 |
| 172 | PG-172 | 9.95 | 6.47 | 75.0 (50.47) | 1232.30 |
| 173 | PG-173 | 10.30 | 6.20 | 59.5 (58.69) | 0981.75 |
| 174 | PG-174 | 9.55 | 5.95 | 73.0 (56.47) | 1130.90 |
| 175 | PG-175 | 7.10 | 5.60 | 69.5 (58.69) | 0882.85 |
| 176 | PG-176 | 8.40 | 5.80 | 73.0 (50.14) | 1036.60 |
| 177 | PG-177 | 9.64 | 5.85 | 57.0(49.00) | 0882.81 |
| 178 | PG-178 | 12.35 | 7.99 | 80.5 (63.79) | 1686.10 |
| 179 | PG-179 | 7.90 | 5.25 | 66.5(64.52) | 0874.54 |
| 180 | PG-180 | 10.45 | 7.97 | 80.0 (49.02) | 1474.03 |
| 181 | PG-181 | 9.40 | 5.60 | 65.0 (54.63) | 0974.90 |
| 182 | PG-182 | 9.70 | 5.60 | 73.0 (53.72) | 1116.60 |
| 183 | PG-183 | 09.80 | 6.50 | 70.5 (58.69) | 1149.10 |
| 184 | PG-184 | 10.35 | 8.06 | 79.5 (57.10) | 1463.64 |
| 185 | PG-185 | 11.25 | 8.18 | 82.0 (64.89) | 1593.69 |
| 186 | PG-186 | 10.50 | 7.55 | 77.0 (66.03) | 1390.40 |
| 187 | PG-187 | 9.70 | 7.15 | 63.0 (52.53) | 1061.05 |
| 188 | PG-188 | 7.20 | 6.47 | 70.5(63.79) | 0964.15 |
| 189 | PG-189 | 11.40 | 8.07 | 79.0 (61.34) | 1538.90 |
| 190 | PG-190 | 7.45 | 7.35 | 70.5 (57.10) | 1043.10 |
| 191 | PG-191 | 7.35 | 7.55 | 74.5 ( 59.67) | 1109.15 |
| 192 | PG-192 | 6.25 | 5.15 | 56.5 (48.73) | 0643.60 |
| 193 | PG-193 | 6.40 | 5.30 | 59.0 (50.18) | 0690.30 |
| 194 | PG-194 | 7.30 | 6.45 | 63.0 (52.53) | 0865.60 |
| 195 | PG-195 | 9.95 | 7.30 | 72.0 (58.05) | 1241.75 |
| 196 | PG-196 | 8.40 | 5.80 | 73.0 (59.67) | 1036.60 |
| 197 | PG-197 | 12.72 | 8.32 | 81.0 (64.15) | 1705.00 |
| 198 | Control | 06.55 | 4.75 | 52.5 (49.89) | 0593.25 |
| 199 | Reference strain:  *Pseudomonas fluorescence* | 13.82 | 8.75 | 85.5(67.61) | 1873.31 |
| S.Em±  CD@ 1% | | 0.23 | 0.18 | 1.74 | 33.94 |
|  |  | 0.65 | 0.51 | 4.85 | 94.74 |

Note

*Figures in the parenthesis indicate arcsine values

**Supplementary Table 3:** Morphological characteristics of the selected rhizobacterial isolates

| **Sl. No** | **Code of the isolates** | **Colony morphology** | | | | | **Cell morphology** | | |
| --- | --- | --- | --- | --- | --- | --- | --- | --- | --- |
|  |  | **colony shape** | **Colour** | **Elevation** | **Consistency** | **Margin** | **Endospore** | **Gram reaction** | **Cell Shape** |
| 1 | PG-145 | Circular | Creamy white | Raised | Dry | Undulated | - | - | Rod |
| 2 | PG-148 | Circular | CreamyWhite | Flat | smooth | Entire | - | - | Rod |
| 3 | PG-152 | Irregular | Creamy white | Flat | Dry | Undulated | + | + | Rod |
| 4 | PG-178 | Circular | Yellowish | Convex | Smooth | Entire | - | - | Rod |
| 5 | PG-197 | Irregular | Yellowish | Convex | Smooth | Entire | - | - | Rod |
| 6 | Reference strain( *P. fluorescens*) | Circular | Creamy  white | Flat | Dry | Entire | - | - | Rod |

**Supplementary Table 4:** Biochemical characteristics of the selected rhizobacterial isolates

| **Sl.**  **No** | **Code of the isolates** | **Biochemical characteristics** | | | | | | | | | | |
| --- | --- | --- | --- | --- | --- | --- | --- | --- | --- | --- | --- | --- |
|  |  | **1** | **2** | **3** | **4** | **5** | **6** | **7** | **8** | **9** | **10** | **11** |
| **1** | PG-145 | **-** | **-** | **-** | **+** | **+** | **+** | **+** | **+** | **+** | **+** | **+** |
| **2** | PG-148 | **+** | **-** | **-** | **+** | **+** | **+** | **+** | **+** | **+** | **+** | **+** |
| **3** | PG-152 | **+** | **-** | **-** | **+** | **+** | **+** | **+** | **+** | **+** | **+** | **+** |
| **4** | PG-178 | **+** | **-** | **-** | **+** | **-** | **+** | **-** | **+** | **-** | **+** | **+** |
| **5** | PG-197 | **+** | **-** | **-** | **+** | **-** | **-** | **-** | **-** | **-** | **+** | **+** |
| **6** | Reference strain (*P. fluorescens)* | **+** | **-** | **-** | **+** | **+** | **+** | **+** | **+** | **+** | **+** | **+** |

Note:

1. Starch hydrolysis 2) Indole production 3) Lactose fermentation 4) Citrate utilization

5) Catalase test 6) Casein hydrolysis 7) Gelatin hydrolysis 8) Nitrate reduction

9) Urease test 10) Oxidase 11) Ammonia production

+: Positive, -: Negative

**Supplementary table 5: Treatment details**

| **Treatment details** | |
| --- | --- |
| T_1_ | PG-145 |
| T_2_ | PG-148 |
| T_3_ | PG-152 |
| T_4_ | PG-178 |
| T_5_ | PG-197 |
| T_6_ | Reference strain (*Pseudomonas fluorescence*) |
| T_7_ | RDF: Recommended Dose of Fertilizer |
| T_8_ | Absolute control |

**Supplementary table 6: Reaction mixture:** Amplification was carried out in a total reaction volume of 20µl with the following components.

| **Components** | **concentration** | **Volume(**µl) |
| --- | --- | --- |
| Taq buffer with Mg^2+^ | 10 X | 2.0 |
| dNTPs | 2.5 mM each | 1.0 |
| Forward primer 27 F | 5 pM | 1.0 |
| Reverse primer 1492 R | 5 pM | 1.0 |
| Taq DNA polymerase | 5 U/µl | 0.2 |
| DNA template | 100 ng/ µl | 1.0 |
| Nuclease free water | - | 13.8 |
| **Total** | - | 20.0 |

**Supplementary table 7: PCR conditions** The PCR reaction mixture was used for the amplification under the following conditions.

| **Cycle** | **Steps** | **Temperature (˚C)** | **Time(min)** | **Cycles** |
| --- | --- | --- | --- | --- |
|  | Lid temperature | 105 | **-** |  |
| I | Initial denaturation | 95.0 | 10 | 1 |
| II | Final denaturation  Annealing  Extention | 94.0  55.0  72.0 | 1  1  1 | 35 |
| III | Final extension  Hold | 72.0  4.0 | 10  30 | 1  - |

After completing specified amplification cycles, the contents were later subjected to Agarose gel electrophoresis.

**Supplementary Figure 1 (a to e): Rapid screening of native rhizobacterial isolates by seed germination test using roll towel method**

Control

*Pseudomonas fluorescens*

**Supplementary Figure 2 (a-e): Antagonistic activity of native rhizobacterial isolates against *Macrophomina phaseolina* a) control plate b) PG-152 strain c) Reference strain d) PG-148 e) PG-145 strain**

*Pseudomonas fluorescens*

**Supplementary Figure 3 (a-c): Antagonistic activity of native rhizobacterial isolates against *Fusarium oxysporum*; a) Control b) Reference strain, and c) PG-152 strain**

**Supplementary Figure 4: Zinc solubilization Supplementary Figure 5: IAA production**

**A. No control B. Strong HCN production C. Weak HCN**

**Supplementary Figure 6 (a-c): HCN Production by selected native rhizobacterial isolates**

**Supplementary Figure 7 (a-b): P solubilization of the selected rhizobacterial isolates**

**Supplementary figure 8: Siderophore production by rhizobacterial isolates**

**A. Gram negative**

**B. Gram positive**

**Supplementary Figure 9: Morphological characteristics of the native rhizobacterial isolates**

**A. Starch Hydrolysis B. Casein hydrolysis**

**C. Citrate utilization**

**Supplementary figure 10 (a-c). Biochemical characterization of native rhizobacterial isolates**


**Supplementary Figure 11: General view of pot culture experiment**

**Supplementary figure 12: Influence of native rhizobacteria on plant height of sorghum under pot culture studies**

**Supplementary figure 13: Genomic DNA of the selected native rhizobacterial isolates**
